# IL-10-STAT3 axis preserves epithelial mitochondrial homeostasis during bacterial challenge

**DOI:** 10.1080/19490976.2026.2661411

**Published:** 2026-04-23

**Authors:** Saranya Navaneetha Krishnan, Amit Jaiswal, Armaan Mohan, Arthur Wang, Timothy E. Shutt, Derek M. McKay

**Affiliations:** a Gastrointestinal Research Group, Inflammation Research Network, Host-Parasite Interactions Program, Department of Physiology & Pharmacology, Calvin, Phoebe and Joan Snyder Institute for Chronic Diseases, Cumming School of Medicine, University of Calgary, Calgary, Alberta, Canada; b Departments of Medical Genetics and Biochemistry & Molecular Biology, Cumming School of Medicine, Hotchkiss Brain Institute, Snyder Institute for Chronic Diseases, Alberta Children's Hospital Research Institute, University of Calgary, Calgary, Alberta, Canada

**Keywords:** Mitochondrial network, mitochondrial dysfunction, interleukin (IL)-10, mtSTAT-3, colonic organoid, epithelium, barrier function, bacterial pathobiont, *E. coli*-LF82

## Abstract

Increasing evidence implicates mitochondrial dysfunction in the pathogenesis of inflammatory bowel disease (IBD), with the IBD-associated pathobiont adherent-invasive *E. coli*-LF82 (AIEC) promoting epithelial mitochondrial depolarization and fragmentation and a concomitant reduction in barrier function. We hypothesized that the anti-inflammatory cytokine interleukin-10 (IL-10), essential for enteric homeostasis, would protect against AIEC-induced mitochondrial disruption. Mitochondrial fragmentation in colonic organoids following infection with *E. coli*-LF82 was reduced by IL-10 pretreatment + cotreatment. IL-10 significantly reduced the *E. coli*-LF82 induced mitochondrial dysfunction in the human colon-derived T84 epithelial cell line as measured by mitochondrial network analysis, membrane potential, permeability transition pore opening, oxidative phosphorylation (via oxygen consumption rate), and epithelial barrier integrity. IL-10-treated T84 cells and organoids exhibited increased phosphorylation of STAT3 at serine 727 (a requirement for STAT3 activity at mitochondria), which was abrogated by pharmacological inhibition of ERK (but not JAK) activation, leading to loss of protection against *E. coli-LF82-induced* mitochondrial dysfunction. Re-expression of wild-type STAT3, but not the S727A mutant, in HT-29 (human colon epithelial cell line) STAT3 knockout cells restored mitochondrial depolarization and ATP levels, underscoring the role of ERK-driven STAT3^S727^ phosphorylation in IL-10's protective mechanism. In contrast, IL-22, which primarily activates STAT3^Y705^ and not STAT3^S727^, failed to prevent AIEC-induced mitochondrial dysfunction. Thus, in the context of exposure to an *E. coli* pathobiont, IL-10 supports gut epithelial homeostasis via ERK-dependent STAT3^S727^ maintenance of mitochondrial integrity, contributing to preservation of epithelial barrier function.

## Graphical abstract

**Figure:**
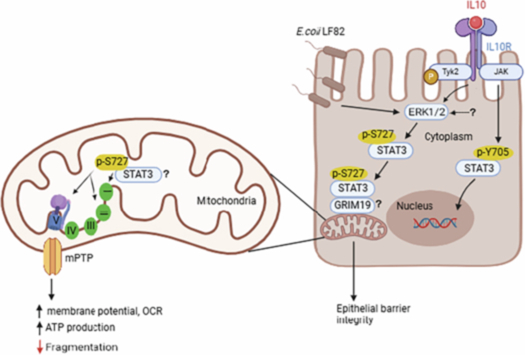

Schematic illustration depicting the proposed mechanism by which IL-10–STAT3 signaling restores mitochondrial function and epithelial barrier integrity during *E. coli*-LF82 infection. Upon IL-10 binding to its receptor (IL10R), JAK and Tyk2 kinases activate STAT3 via phosphorylation at tyrosine residue 705 (p-Y705), leading to its nuclear translocation. Concurrently, *E. coli*-LF82 and IL-10 activate MAPK-dependent phosphorylation of STAT3 at serine residue 727 (p-S727), promoting its mitochondrial function, likely via GRIM19 and components of the electron transport chain. An additional, yet unidentified IL-10-independent signal may be necessary to fully engage this mitochondrial protective pathway. Mitochondrial p-STAT3^S727^ supports oxidative phosphorylation (oxygen consumption rate), ATP production, and membrane potential while limiting mitochondrial permeability transition pore (mPTP) opening and excessive fragmentation that correlate with preservation of epithelial barrier integrity in response to bacterial infection.

## Introduction

Since Roediger posited that ulcerative colitis may be a metabolic disorder,^1^ evidence has accumulated illustrating mitochondrial abnormalities in intestinal tissue from individuals with inflammatory bowel disease (IBD).^2-4^ These findings have been complemented by *in vitro* work and *in vivo* murine studies implicating mitochondrial dysfunction in the initiation, exaggeration, or reactivation of intestinal inflammation.^5-7^

Mitochondria exist in a dynamic network that continuously undergoes remodeling via fission and fusion processes to regulate cell homeostasis and bioenergetics.^8^^,^^9^ Recently, fragmentation of the epithelial mitochondrial network was described in IBD-biopsies and tissue from mice with di-nitrobenzene sulfonic acid (DNBS)-induced colitis: the extent of mitochondrial fragmentation correlated with disease severity.^10^ Notably, systemic treatment with the mitochondrial anti-fission drug P110 reduced the severity of disease in the DNBS and dextran sodium sulfate (DSS) murine models of colitis.^7^ In addition, bacterial pathobionts are increasingly identified in colitis,^11-14^ and the prototypic pathobiont in Crohn's disease, adherent-invasive *E. coli* (AIEC: strain LF82), caused mitochondrial membrane depolarization, mitochondrial fragmentation, and ATP depletion in human colon-derived epithelial cell lines and organoids.^15^^,^^16^ Collectively, these studies suggest that epithelial mitochondrial dynamics can be perturbed in IBD and hence may be a new therapeutic target.^17^^,^^18^

Interleukin-10 (IL-10) plays crucial roles in immunomodulation and the maintenance of gut homeostasis.^19^^,^^20^ Mice deficient in IL-10 develop enterocolitis, and loss-of-function mutations in the IL-10 receptor cause severe, early-onset IBD.^21^^,^^22^ The IL-10 receptor is expressed on various cell types, including enteric epithelial cells, where IL-10 signaling is primarily thought to be mediated through activation of signal transducer and activator of transcription 3 (STAT3) via phosphorylation at tyrosine 705 (*p*-STAT3^Y705^).^23^^,^^24^ This canonical phosphorylation event promotes STAT3 dimerization, nuclear translocation, and transcription of anti-inflammatory genes. Given the well-established transcriptional role of *p*-STAT3^Y703^ well established, recent evidence suggests that IL-10 can support mitochondrial function through noncanonical pathways involving mitochondrial STAT3 (mtSTAT3), a regulator of mitochondrial respiration and cellular energy metabolism. STAT3 translocation into mitochondria can be facilitated through interaction with GRIM-19, a subunit of mitochondrial Complex I.^25^ Phosphorylation at serine727 enhances STAT3's binding to GRIM-19, thereby promoting mitochondrial import.^25^ Within mitochondria, STAT3 interacts with components of the electron transport chain, particularly complexes I and II, modulating respiration and reactive oxygen species (ROS) production.^25^ In addition, mtSTAT3 regulates the mitochondrial permeability transition pore (mPTP), contributing to the maintenance of mitochondrial membrane potential and ATP synthesis.^26^ These findings indicate that IL-10-mediated signaling extends beyond classical nuclear transcription to regulate mitochondrial homeostasis via STAT3-dependent mechanisms.

STAT3 S727 phosphorylation is mediated by kinases such as ERK1/2, mTOR, and CDK5, with ERK1/2 playing a prominent role in stress and cytokine responses.^27-29^ In macrophages, IL-10 enhances mitochondrial fitness by promoting oxidative phosphorylation, stimulating mitophagy of damaged mitochondria, and limiting inflammatory activation, including suppression of the NLRP3 inflammasome.^30^ These data raise the possibility that IL-10 could preserve epithelial mitochondrial structure and function in response to bacterial challenges. We find that IL-10 markedly reduced mitochondrial fragmentation in *E. coli-*LF82-infected human colon epithelial cells. This protective effect required STAT3 S727 phosphorylation and was dependent on ERK1/2 signaling, linking upstream ERK activation to STAT3-mediated mitochondrial preservation. These data uncover another important contribution of IL-10 to normal gut function (e.g., maintenance of epithelial mitochondrial function in the context of infection with a bacterial pathobiont), which presents a new therapeutic possibility to preserve epithelial function in defined cohorts of individuals with IBD.

## Methods

### Cell culture

The human colon-derived T84 cell line (originally from an adult male: passages 60–110) was cultured in a 1:1 Dulbecco's modified Eagle medium/F12 Ham medium (Sigma Chemical Co., St. Loius, MO) containing HEPES (2 mmol/L, Sigma), L-glutamine (2.68 mmol/L; Gibco, MA), sodium pyruvate (0.6 mmol/L; Sigma), sodium bicarbonate (0.015%; Gibco), and penicillin (120 U/mL)-streptomycin (0.12 mg/mL, Sigma) and 10% (vol./vol.) fetal bovine serum (FBS: Thermofisher), and maintained under 5% CO_2_ at 37 °C. The human colon-derived HT-29 cell line (originally from an adult female: passages 40–60) and STAT3 knockout (KO) HT-29 cells were cultured in DMEM (#D5796, Sigma) containing nonessential amino acids (100x, M7145, Sigma), penicillin‒streptomycin and 5% FBS, and maintained under 5% CO_2_ at 37 °C.

Human colonic organoids were obtained from the Human Organoid Innovation Hub, Snyder Institute of Chronic Diseases, University of Calgary, under ethics approval REB-18-0631. Healthy donor information is provided in Table 1. Enterocytes were isolated, cultured, and differentiated as previously described.^31^ Experiments were performed on passage 4-24 organoid 2D-monolayers. Adherent invasive *E. coli* (AIEC) strain LF82 was maintained on Columbia sheep blood agar and cultured in antibiotic broth (#70184; Fluka Analytical).^32^

**Table 1.** Information on organoids derived from healthy donors.

Healthy donor sample ID	Age/Sex	Region
HC3020-102	51/F	Descending colon
HC4993-186	59/M	Transverse colon
HC4354-880	21/F	Transverse colon
HC5241-616	79/F	Colon (unspecified)

### Treatment with cytokines, inhibitors for JAK and ERK, and *E. coli-*LF82

Since many cells in the gut produce IL-10, we tested two experimental paradigms: (i) an 18 h pretreatment and (ii) 18 h pretreatment plus cotreatment long with *E. coli* (hereafter referred to as pre/cotreatment). [IL-10 acts in a time-dependent manner, with pretreatment for up to 24 h used to restore mitochondrial function, including improved membrane potential, and increased ATP production.^30^^,^^33-35^ Pilot time-course experiments showed that a pretreatment of 12 h or greater was needed to reverse mitochondrial fragmentation caused by exposure to *E. coli*-LF82 (Suppl. Figure 1). Thus, our rationale is to employ 18 h pretreatment + IL-10 cotreatment at the time of addition of *E. coli*-LF82 to the culture.] T84 cells (10^6^/mL) were seeded and two days later were treated with human recombinant IL-10 or IL-22 (both 10 ng/mL; from Cedarlane, Ontario, CA) and 18 h later infected with *E. coli-*LF82 (10^8^ colony forming units (cfu) for a multiplicity of infection (MOI = 100) for 4 h^15^ along with IL-10 or IL-22 at 37 °C. For pharmacological inhibitor studies, epithelia were exposed to the Janus Kinase (JAK) inhibitor tofacitinib (10 μM)^36^ or the ERK inhibitor, PD-98058 (10 μM)^37^ for 1 h, followed by IL-10 for 18 h and then IL-10 + *E. coli-*LF82 (Suppl. Figure 1A). Inhibitors were present throughout the experiment.

### Live cell imaging

To examine mitochondrial morphology, T84 cells seeded on 8-well chamber slides (Lab-Tek, 155409; ThermoFisher Scientific) were treated as per the experimental design (Suppl. Figure 1A) and then stained with MitoTracker Red (100 nM, Invitrogen Detection Technologies, Molecular Probes, MA) for 30 min at 37 °C, washed, stained with Hoescht dye (1 mg/mL for 15 min; ThermoFisher), washed, and treated with *E. coli-*LF82 (MOI = 100) for 4 h. Images were captured on a Olympus spinning disk confocal microscope (SD-OSR) with 100× magnification (the microscope settings were adjusted to 180 ms for the SD-561 red laser to detect Mitotracker Red and 150 ms for the SD-405 blue laser to capture the Hoechst signal). Imaging of the nuclei was used to randomly select epithelial cells for mitochondrial analysis. Twenty cells per epithelial preparation were assessed blindly, and mitochondrial network morphology was defined as fused, fragmented, or intermediate as previously described.^15^

Mitochondrial membrane potential was assessed in epithelial cells cultured on 8-well chamber slides. Cells were stained with tetramethyl rhodamine ethyl ester (TMRE, 400 nM; ThermoFisher) for 30 min at 37 °C, followed by Hoechst staining. Imaging was performed using an Olympus spinning disk confocal microscope (SD-OSR) with 100× magnification. The microscope was set to 180 ms for the SD-561 red laser and 150 ms for the SD-405 blue laser to detect TMRE and Hoechst signals, respectively. Carbonyl cyanide 4-(trifluoromethoxy) phenylhydrazone (FCCP; 10 μM for 30 min) was used as a positive control to induce the loss of mitochondrial membrane potential.

### Mitochondrial fragmentation count and branch length analysis

Briefly, quantitative analysis was performed using ImageJ, where networks were skeletonized, background signals removed, and mitochondrial particles counted. The mitochondrial fragment count (MFC) was calculated as the number of particles per unit area, with fused networks exhibiting fewer, larger particles compared to fragmented networks.^16^^,^^38^ Mitochondrial branch lengths were quantified using automated skeletonization in ImageJ (version 1.54f). Individual cells were delineated by manually drawing regions of interest (ROIs) to enable per-cell measurements. Raw fluorescence images were converted to 8-bit and pre-processed using despeckle filtering and outlier removal to minimize background noise. Binary images were generated using Otsu automated thresholding and subsequently skeletonized. Branch lengths were measured with the Analyze Skeleton (2D/3D) plugin, which traces each mitochondrial branch and reports its length in calibrated units (µm).^39^ For area measurements, the total mitochondrial area within each ROI was determined from the binary masks using the “Analyze → Measure” function, yielding calibrated area values (µm²) for each cell.^39^

### Immunofluorescence

Human colonic organoids and T84 cells were grown on 3-μm filter supports (Corning, ThermoFisher) and coverslips, respectively, for immunostaining of fixed cells.^10^ Following treatment, the cells were fixed with 4% paraformaldehyde for 15  min, washed, permeabilized with 0.2% Triton X-100 for 15 min, and blocked with 10% goat serum for 1 h at room temperature (RT). The cells were incubated with anti-TOMM20 antibody (ab186735, 100 μg/mL, Abcam, Cambridge, UK) or anti-ZO-1 antibody (61–7300, 50 μg/mL, Invitrogen) at 4 °C overnight, washed, and then incubated with goat anti-rabbit secondary antibody conjugated with Alexa fluor 568 (A11011, 4 μg/mL, ThermoFisher) for 2 h at RT. The cells were then washed and stained with 4′,6-diamidino-2-phenylindole (DAPI, 10 μg/mL; ThermoFisher) for 10 min, washed and mounted with Dako fluorescent mounting medium (Dako North America Inc., Agilent Technologies). The images were taken on an Olympus spinning disk confocal microscope at 100× magnification. Twenty cells per epithelial preparation were assessed on coded slides and mitochondrial network morphology was assessed.

### Flow cytometry

To assess mitochondrial membrane potential, epithelial cells (10⁶/mL) were seeded in 6-well plates and subjected to the indicated treatments. Cells were incubated with TMRE (400 nM) for 30 min at 37 °C, followed by gentle washing. Subsequently, cells were detached using Accutase (SCR005, Millipore, Burlington, MA, USA) for 15 min, and the reaction was neutralized with T84 culture medium supplemented with 10% FBS. The cell suspension was passed through a 100 µm cell strainer (#22363549; ThermoFisher), stained with DAPI (10 μg/mL) for 5 min to exclude nonviable cells, and immediately analyzed by flow cytometry using a BD FACS Canto cytometer (BD Biosciences, Franklin Lakes, NJ) operated with BD FACSDiva software. TMRE fluorescence was detected using the PE laser (561 nm), and DAPI was detected using the BV421 laser (405 nm). Cells treated with the mitochondrial uncoupler, FCCP (10 μM), served as positive controls for membrane depolarization. Mitochondrial membrane potential was quantified as the percentage change in TMRE mean fluorescence intensity (MFI) relative to untreated controls, with a decrease in MFI indicating increased depolarization. Flow cytometry data were analyzed using FlowJo software (BD Biosciences).^40^

To evaluate mPTP opening, T84 cells were seeded in 6-well plates, subjected to the indicated treatments, and detached using Accutase. Cells were then incubated with calcein-AM (1 µM; #M34153, ThermoFisher) ± CoCl_2_ (1 mM) in Hanks' balanced salt solution (HBSS) for 15 min at 37 °C in a 5% CO₂ incubator. Following incubation, cells were washed and resuspended in HBSS and stained with DAPI. Samples were analyzed by flow cytometry using a BD FACS Canto cytometer. Calcein fluorescence was detected using the FITC laser (488 nm) and DAPI was detected using the BV421 laser (405 nm).^41^

### Bacterial internalization

T84 cells (10^6^/ml) were seeded on 6-well plates and 4 h after exposure to *E. coli*-LF82, 10 μL of culture medium was collected to determine the number of extracellular bacteria. The epithelia were then washed with PBS and treated with gentamycin (100 μg/mL) for 1 h. The cells were then lysed with 1% Triton X100, and the lysate was cultured for 18 h at 37 °C on blood agar plates. Colony-forming units (CFU) were counted and the data presented as the percentage of internalization.^15^

### Epithelial barrier function

Human colonic organoids (0.5 × 10^6^ cells) or T84 cells (10^6^/ml) were cultured on Transwell filter supports (3 μm pore size) until transepithelial resistance (TER), measured with a voltmeter and matched chopstick electrodes, reached ≥1200 Ω × cm²: TER was recorded before and 5 h after infection with *E. coli*-LF82. To assess bacterial translocation, 200 μL of medium from the basal compartment was collected at 5 h (T84 monolayers) or 11 h (organoid monolayers), centrifuged at 15,000× g for 10 min, and the pellets were resuspended in 200 μL of PBS. Samples were serially diluted and plated on blood agar for 18 h at 37 °C to determine CFUs. The percentage translocation relative to the *E. coli*-LF82 infection was calculated by dividing the number of CFUs recovered from IL-10-treated cells by the number of CFUs recovered from infected cells (without IL-10 treatment) and multiplying by 100.^15^

### Cytokine measurements

IL-8 production by T84 cells, a characteristic of *E. coli*-LF82-infected cells, and IL-10 levels were assessed in duplicate samples by commercial ELISA, with lower limits of detection of 9 pg/mL, following the manufacturer's instructions (R&D Systems Inc.).

### Total RNA isolation and quantitative PCR

RNA was extracted from T84 cells using the RNeasy Mini Kit (Qiagen), and RNA quality and quantity were verified with a NanoDrop (NanoDrop 1000 Spectrophotometer, Thermo Fisher Scientific, Wilmington, DE). One microgram of RNA was converted to cDNA using the I-Script kit (Bio-Rad). qPCR was performed on a StepOnePlus system using SYBR Green Supermix (Bio-Rad, Cat. # 1725274) with 0.5 µg of cDNA and primers (the primer sequences for human SOCS3 and human 18S were as follows: hSOCS3: forward 5′-CATCTCTGTCGGAAGACCGTCA-3′ and hSOCS3: reverse 5′-GCATCGTACTGGTCCAGGAACT-3′ and h18S: forward 5′-ACGCGCGCTACACTGACTGG-3′ and h18S reverse: 5′-CGATCCGAGGGCCTCACTAAACC-3′ (Invitrogen)). CT values were normalized to 18S and comparative quantification was determined using the ΔΔCT method and expressed relative to controls.^42^

### Mitochondrial respiration

Mitochondrial oxygen consumption rate (OCR), used as a surrogate measure of oxidative phosphorylation, was assessed using the XF24 Extracellular Flux Analyzer (Agilent Technologies) according to the manufacturer's protocol. T84 cells (2 × 10⁵ per well) were seeded into XF24 cell culture microplates and subjected to the indicated treatments. After 4 h of *E. coli*-LF82 infection and respective treatments, cells were washed and incubated in Seahorse XF assay medium (supplemented with 25 mM glucose, 4 mM L-glutamine, and 2 mM sodium pyruvate, pH 7.4). Mitochondrial stress testing was performed by sequential injection of oligomycin (1 µM), FCCP (1 µM), antimycin A (1 µM), and rotenone (1 µM). Upon completion of the assay, cells were lysed using NP-40 buffer (50 mM Tris-HCl pH 8.0, 150 mM NaCl, 1% NP-40, 1 mM sodium orthovanadate) containing a protease inhibitor cocktail and total protein content per well was quantified using the Bradford assay (Bio-Rad, CA). Briefly, lysates were incubated with Bradford reagent according to the manufacturer's instructions, and absorbance was measured at 595 nm using a microplate reader. A standard curve generated using bovine serum albumin (BSA) was used to calculate protein concentration. OCR values were normalized to total protein content per well for accurate comparison across samples.^40^

### ATP assay

T84 cells (5 × 10^5^) were seeded on 12-well plates, and after treatment, the total cellular ATP level was determined using CellTiter-Glo Luminescent Cell Viability Assay (Promega, Madison, USA) as per the manufacturer's protocol. The luminescence was measured using flat-bottom, white-walled, 96-well plates in a Victor3V 1420 Multilabel Counter (PerkinElmer). A standard curve was generated to calculate ATP concentration, and the data were normalized to total protein concentration (Bradford assay).^15^

### Subcellular fractionation

Mitochondria were isolated from T84 cells using Percoll gradient centrifugation, as previously described.^43^ Cells were homogenized in 10 mL of IBcells-1 buffer (225 mM mannitol, 75 mM sucrose, 0.1 mM EGTA, 30 mM Tris-HCl, pH 7.4). The homogenate (H) was centrifuged at 600* *g for 5 min at 4 °C to separate the nuclear fraction (*N*). The resulting supernatant was further centrifuged at 7,000*× *g for 10 min at 4 °C the cytosolic fraction (C). The pellet was resuspended in 10 mL of IBcells-2 buffer (225  mM mannitol, 75 mM sucrose, 30 mM Tris-HCl, pH 7.4) and centrifuged again (7,000 g, 10 min, 4 °C). The pellet, containing the mitochondria was resuspended in 10 mL of IBcells-2 buffer and centrifuged (10,000* *g, 10 min, 4 °C). The final pellet was resuspended in 2 mL of mitochondria resuspension buffer (MRB: 250 mM mannitol, 0.5 mM EGTA, 5 mM HEPES, pH 7.4) and layered over a discontinuous gradient consisting of 8 mL Percoll medium (30% Percoll, 225 mM mannitol, 25 mM HEPES, 1 mM EGTA, pH 7.4) and 4 mL MRB. The gradient was centrifuged at 95,000 g for 30 min at 4 °C. Following centrifugation, pure mitochondria (Mt) were collected from the bottom of the tube. All fractions were washed and centrifuged at 6,300 g for 10 min at 4 °C for final purification.

### Western blot

Whole cell lysates (100 µg) in NP-40 buffer (50 mM Tris-HCl, pH 8.0, 150 mM NaCl, 1% NP-40, 1 mM sodium orthovanadate) with a protease inhibitor cocktail were resolved in 10% SDS polyacrylamide gels and electroblotted onto nitrocellulose membranes. Membranes were blocked in 5% BSA then incubated with the indicated primary antibody: pSTAT3^S727^ (9134S, Cell Signaling, Boston, MA, USA, 2 μg/mL), pSTAT3^Y705^ (9131S, Cell Signaling, 2 μg/mL), STAT3 (9139S, Cell Signaling, 2 μg/mL), p42/44 MAPK (9102, Cell Signaling, 2 μg/mL), the voltage-dependent anion channel (VDAC) (B-6, Santa Cruz Biotech, Dallas, TX, USA, 0.4 μg/mL), proliferating cell nuclear antigen (PCNA) (PC-10, Santa Cruz Biotech, 0.4 μg/mL), lactate dehydrogenase (LDH-A) (E-9, Santa Cruz Biotech, 0.4 μg/mL) and GAPDH (6C5, Abcam, Cambridge, United Kingdom, 2 μg/mL) at 4 °C overnight. After washing with tris-buffered saline (TBS) + Tween-20 (TBST) (50 mM Tris-HCl, pH 7.6, 0.1% Tween-20, 0.8% NaCl), membranes were incubated with horseradish peroxidase-conjugated secondary anti-rabbit (SC2357, Santa Cruz, 80 ng/mL) or anti-mouse antibody (SC2031, Santa Cruz, 80 ng/mL) for 2 h. Immunoreactive bands were detected using Clarity Max western blot ECL reagent (Bio-Rad). The intensity of protein bands was quantified using Image J and normalized to the corresponding total protein levels.

### Generation of STAT3 knockout HT-29 cells

STAT3 knockout (STAT3-KO) HT-29 cells were generated at the Center for Genome Engineering, University of Calgary, using CRISPR-Cas9 genome editing. A short guide RNA (sgRNA) targeting exon 22 of the human *STAT3* gene was designed, synthesized, and cloned into the pX458 vector, followed by confirmation through DNA sequencing. The *STAT3* sgRNA construct was transfected into HT-29 cells using jetOPTIMUS transfection reagent according to the manufacturer's instructions. GFP-positive cells were single-cell sorted into 96-well plates by flow cytometry and expanded in culture. Clones were screened for STAT3 disruption and loss of STAT3 protein expression was validated by Western blot analysis.

### Retroviral transduction

HT-29 STAT3-KO cells expressing WT-STAT3 and S727A-STAT3 were generated using retroviral transduction without antibiotic selection. Briefly, Phoenix cells were transfected with plasmids (5 μg) encoding WT-STAT3 (addgene, #71450) or S727-STAT3 (addgene, #71446) using Lipofectamine 3000 (Invitrogen, Carlsbad, CA) as per the manufacture's protocol. Sixteen hours post-transfection, Phoenix cell media was refreshed, and virus-containing supernatant was collected 48 h (day 1) and 72 h (day 2) post-transfection. Virus-containing supernatant was filtered through 0.4 µM PES filters before being aliquoted and frozen at −80°C. HT-29 STAT3-KO cells were transduced using 1:1 virus-containing supernatant and complete media containing polybrene at 8 µg/mL, twice 8 h apart, using day 1 viral supernatant. This transduction method was repeated the next day using day 2 viral supernatant and assessed the expression 72 h after the final infection by immunoblotting.

### Data presentation and analysis

Data are presented as mean ± standard error of the mean (SEM) with “n” representing the number of epithelial monolayers analyzed, based on a specified number of independent experiments. Statistical analyzes were performed using GraphPad Prism software (version 9.3.1). For parametric data, either one-way or two-way ANOVA was used as appropriate, followed by Tukey's multiple comparisons post hoc test. Non-parametric data were analyzed using the Kruskal–Wallis test followed by Dunn's multiple comparisons test or the Mann‒Whitney test. A *p*-value of <0.05 was considered statistically significant. Statistical analysis was not performed when *n* = 3.

## Results

### IL-10 stabilizes the mitochondrial network in AIEC-infected human colonic organoids and intestinal epithelial cells

**Figure 1.**
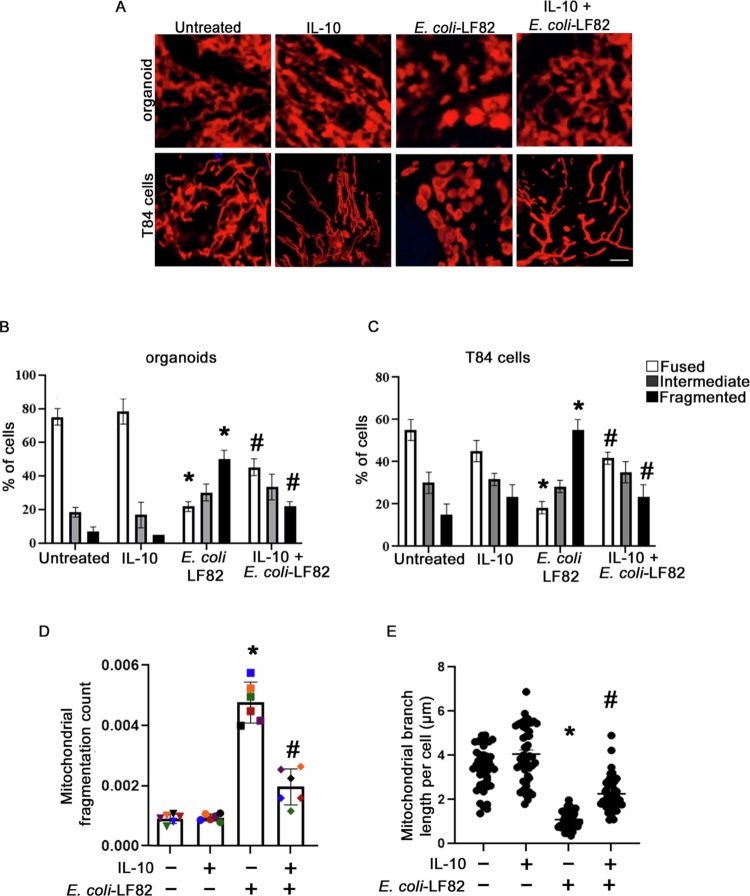
IL-10 prevents epithelial mitochondrial fragmentation evoked by *E. coli*-LF82. (A) Representative confocal microscopy images of mitochondrial networks in control human colonic organoids (top panel) and T84 epithelial cells (bottom panel) and pre/cotreated with IL-10 (10 ng/mL, 18 h pretreatment and cotreatment with *E. coli*) and *E. coli*-LF82 (10^8^ CFU/mL, 4 h). The cells were stained with MitoTracker red and Hoest dye and images were acquired using a ZEISS microscope under 100x oil immersion. Scale bar = 1 µm. Quantification of fused, intermediate, and fragmented mitochondria in human colonic organoids (B; *n* = 6 monolayers from 3 independent experiments) and T84 cells (C: *n* = 6 epithelial monolayers (20 cells/monolayer) from 3 independent experiments) subjected to the indicated treatments (data are mean ± SEM. * and # indicate statistically significant difference at *p* < 0.05 compared to untreated and *E. coli*-LF82-treated cells, respectively, analyzed by two-way analysis of variance (ANOVA) followed by the Tukey multiple comparison test. (D) Mitochondrial fragmentation count and (E) mitochondrial branch length were calculated using Image J applied to randomly selected images from T84 cell monolayers (*n* = 6 epithelial monolayers (20 cells/monolayer) from 3 independent experiments; data are mean ± SEM. * and #, *p* < 0.05 compared to untreated and *E. coli*-LF82-treated cells, respectively, analyzed by One-way ANOVA followed by the Tukey multiple comparison test.

We reported that the pathobiont-adherent invasive *E. coli-*LF82 (10^8^ CFU/mL for 4 h), but not the commensal *E. coli* strain-F18, caused mitochondrial fragmentation, depolarization, and reduced ATP levels in the T84 epithelial cell line.^15^ We confirmed these findings (Suppl. Figure 2A and B) and showed internalized *E. coli*-LF82 in cells with a fragmented mitochondria network (Suppl. Figure 2C). Human colonic organoids and T84 cells pre/cotreated with IL-10 (10 ng/mL, 18 h) and *E. coli*-LF82 (10^8^ CFU/mL, 4 h) displayed significantly reduced *E. coli* LF82-evoked mitochondrial fragmentation (Figure 1A, top panel): 52% of cells assessed in organoids exposed to *E. coli*-LF82 had obvious mitochondrial fragmentation, with only 22% of the cells in the IL-10+*E. coli*-LF82 group having a fragmented mitochondrial network (Figure 1B). These findings were recapitulated in T84 epithelial cells, where IL-10 pre/cotreatment resulted in a less fragmented mitochondrial network compared to *E. coli*-LF82 only infected cells (Figure 1A bottom panel and Figure 1C). Similarly, quantitative analysis of the mitochondrial network in *E. coli*-LF82 infected T84 cells revealed a higher mitochondrial fragmentation count and shorter mitochondrial branch length than non-infected cells and this was significantly improved by IL-10 treatment (Figure 1D and E). IL-10 used as pre-treatment (Suppl. Figure 1) or a co-treatment only (data not shown) did not reduce the T84 epithelial mitochondrial fragmentation caused by *E. coli*-LF82 infection and so neither approach was not adopted. Based on pilot time-course experiments (Suppl. Figure 1), an 18 h pretreatment + cotreatment regimen was selected for subsequent studies.

**Figure 2.**
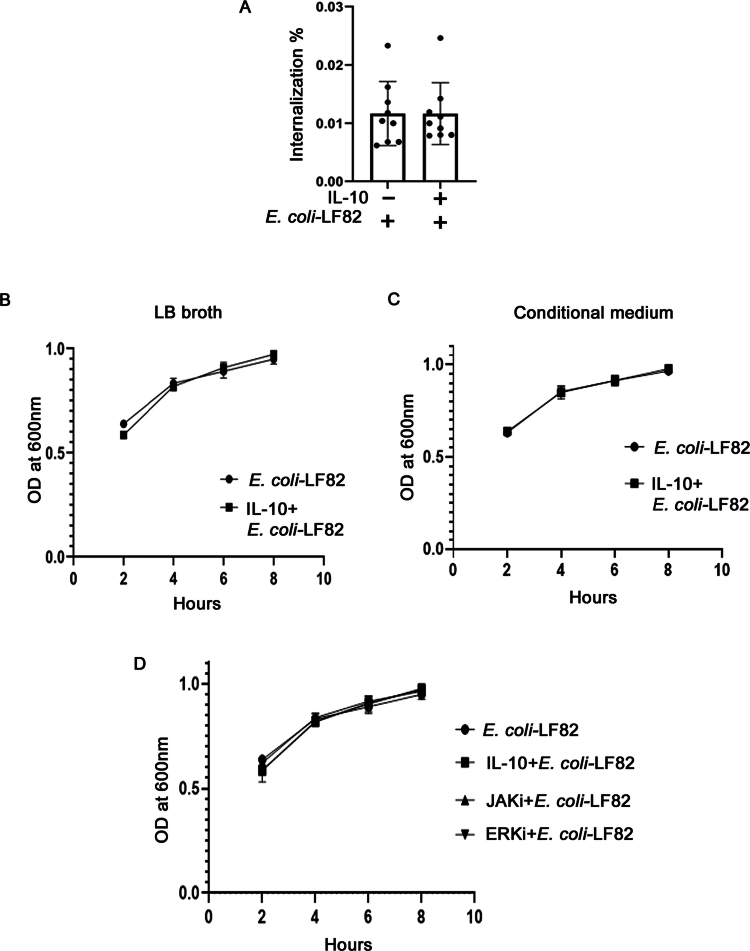
IL-10 affects neither *E. coli*-LF82 invasion into epithelia nor its growth. (A) Bacterial invasion of *E. coli*-LF82 (10^8^ CFU/mL, 4 h; MOI = 100) into T84 epithelial cells ± pre/co-treatment with IL-10 (10 ng/mL, 18 h) (data are mean ± SEM; *n* = 9 epithelial monolayers from 3 separate experiments). Growth of *E. coli*-LF82 (10^4^ CFU/mL) was determined at 2, 4, 6, and 8 h following direct exposure to recombinant IL-10 in LB broth (B) or conditional media retrieved from cultures of IL-10 + T84 cells diluted 1:1 in fresh medium (C), and measuring OD at 600 nm. (D) Growth curve analysis of *E. coli*-LF82 (10^4^ CFU/mL) with IL-10, the JAK inhibitor, tofacitinib (10 μM), or the ERK inhibitor, PD-98058 (10 µM), shows that growth of the bacteria was unaffected by these drugs (*n* = 2–3 growth curves).

To test the possibility that the decrease in mitochondrial fragmentation following IL-10 exposure was due to changes in the growth, attachment and/or invasion potential of *E. coli*-LF82, bacterial epithelial internalization and bacteria growth curves in the presence of IL-10 or culture medium from T84 cell + IL-10 cultures were performed; the latter assessing if the IL-10 induced an epithelial antibacterial response. Interleukin-10 did not directly affect *E. coli*-LF82 invasion of T84 cells (Figure 2A). Neither human recombinant IL-10 (Figure 2B) nor conditioned medium from T84 cells cultured with IL-10 (10 ng/mL for 18 h) (Figure 2C) were bactericidal or bacteriostatic.

Having defined the ability of IL-10 to reduce mitochondrial fragmentation in human organoids and an epithelial cell line, subsequent functional and mechanistic studies concentrated on T84 cells and the HT-29 human colonic epithelial cell line, where transfection efficacy was greatest.

### IL-10 protects mitochondrial function in AIEC-infected epithelia

**Figure 3.**
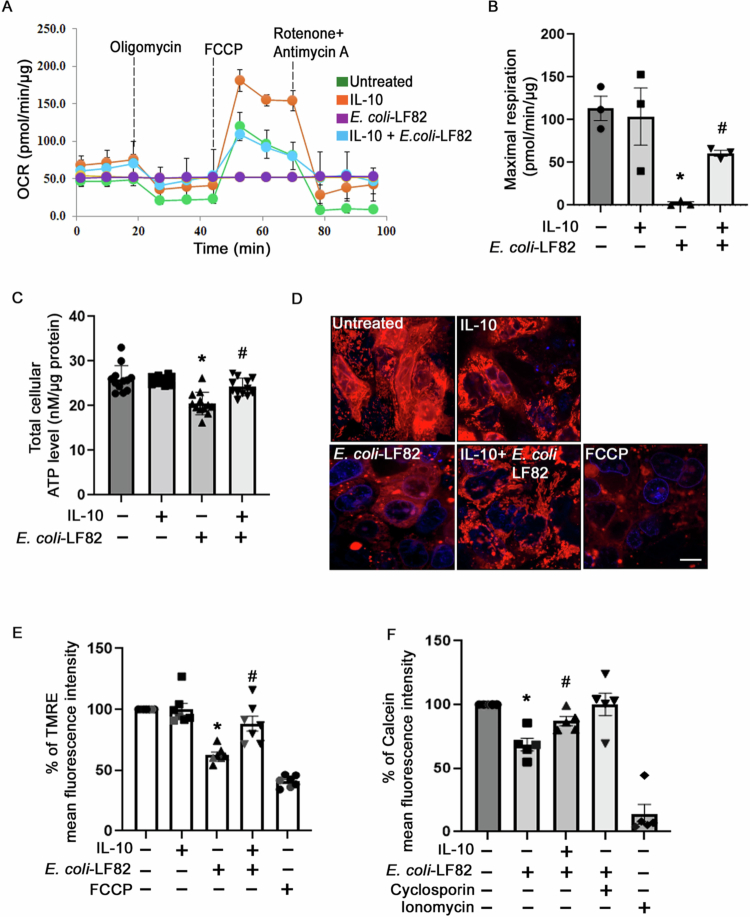
IL-10 prevents T84-epithelial mitochondrial dysfunction evoked by *E. coli*-LF82. Analysis of oxygen consumption rate (A) and quantification of maximal respiration (B) in IL-10 (10 ng/mL, 18 h pretreatment and cotreatment with bacteria)-treated T84 cells exposed to *E. coli*-LF82 (10^8^ CFU/mL, 4 h; MOI = 100) (*n* = 3 epithelial preparations from independent experiments). (C) Total cellular ATP levels in T84 cells subjected to the indicated treatments (*n* = 12 epithelial monolayers from 4 independent experiments). (D) Representative confocal images of mitochondrial membrane potential in T84 cells stained with the fluorescent probe tetramethyl rhodamine ethyl ester (TMRE; 400 nM; FCCP at 4 μM for 30 min used as a positive control; *n* = 6 epithelial monolayers from three experiments; scale bar = 5 µm). (E) Quantification of mean fluorescence intensity of TMRE by flow cytometry (*n* = 7 epithelial preparations from 7 independent experiments). (F) Flow cytometry analysis of mitochondrial calcein-AM (1 μM) fluorescence in T84 cells to measure mitochondrial permeability transition pore (mPTP) opening (cyclosporin A, an inhibitor of mPTP (10  μM for 24 h) and ionomycin (0.5 μM, for 15 min), an inducer of mPTP opening, were used as negative and positive controls; *n* = 5 epithelial monolayers from three independent experiments). Data are mean ± SEM. * and # indicate statistically significant difference at *p* < 0.05 compared to untreated and *E. coli*-LF82-treated cells, respectively, analyzed by one-way ANOVA followed by the Tukey multiple comparison test (B, C) or by the Kruskal‒Wallis test followed by Dunn's multiple comparison (E, F).

Since mitochondrial dysfunction is linked to mitochondrial fragmentation, the ability of IL-10 to prevent or reduce mitochondrial dysfunction in *E. coli*-LF82-infected T84-epithelia was tested. Analysis of oxygen consumption revealed that *E. coli*-LF82 completely shut down mitochondrial OxPhos in T84 cells, which correlated with reduced ATP levels and loss of mitochondrial polarization (Figure 3A–E). Treatment with IL-10 wholly or partially prevented these epithelial mitochondrial abnormalities caused by infection with *E. coli*-LF82.

Opening of the mitochondrial permeability transition pore (mPTP) can lead to mitochondrial depolarization, fragmentation, and reduced respiration. Calcein-AM staining followed by quenching with CoCl_2_ and flow cytometry was used to assess mPTP opening in T84-epithelia. T84 cells infected with *E. coli*-LF82 showed a significant reduction in the % of calcein^+^ cells, indicating increased mPTP opening and this was significantly reduced in IL-10 pre/cotreated epithelia (Figure 3F). The use of cyclosporin A, an mPTP inhibitor, completely prevented the opening of the mPTP in *E. coli*-LF82-infected epithelia (ionomycin used as a positive control) (Figure 3F).

### IL-10 preserves epithelial permeability barrier function in AIEC-infected epithelia

**Figure 4.**
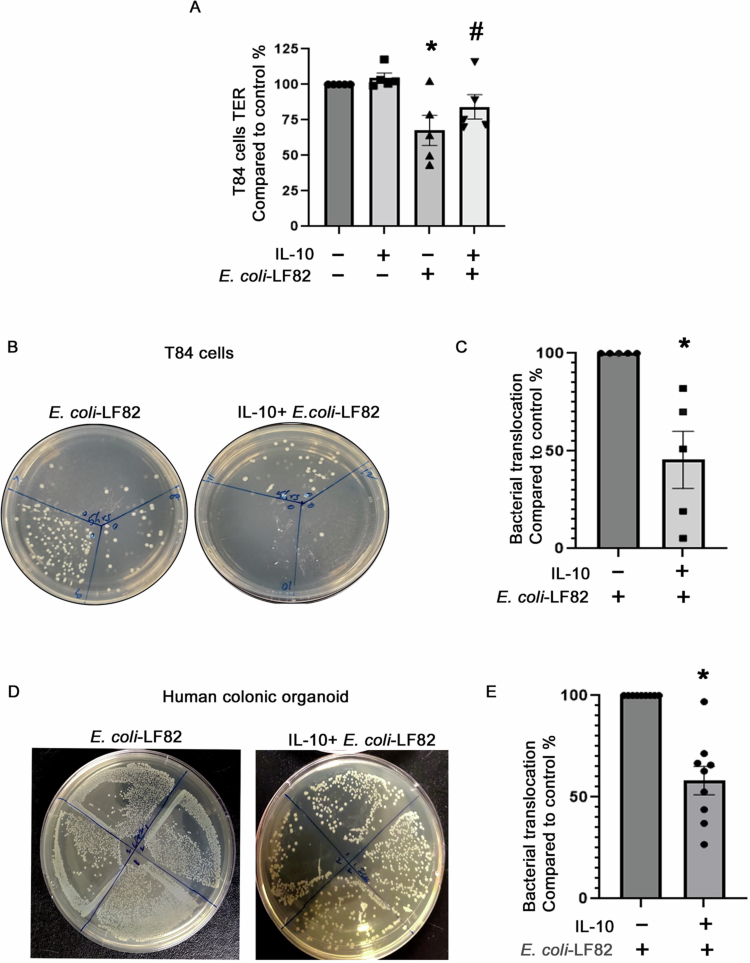
IL-10 protects barrier function in *E. coli*-LF82-infected epithelial cells. (A) The drop in T84 cell monolayer transepithelial resistance (TER) observed 5 h after infection with *E. coli*-LF82 (10^8^ CFU/ml; MOI = 100) was significantly reduced in the presence of IL-10 (10 ng/mL, 18 h pretreatment and cotreatment with bacteria). Baseline starting TER range 1,000–1,200 Ω.cm^2^. (B) Representative images of bacteria colony growth from 200  μL culture medium samples retrieved from the basal compartment of the culture well of T84 cell monolayers and quantified as relative to *E. coli*-only (5 h) in panel C (*n* = 5, five independent experiments (3 replicates/experiment)). Each segmented plate represents individual biological replicate monolayers within the indicated treatment groups. (D) Representative images of bacteria colony growth from culture medium samples retrieved from basal compartment of the culture well of monolayers of human healthy organoids 11 h post-infection with *E. coli* ± per/co-treatment with IL-10 and quantified as relative to *E. coli*-only in panel E (*n* = 9 organoid monolayers from three independent experiments). Data are mean ± SEM; * and # *p* < 0.05 compared to control and *E. coli*-LF82, respectively, analyzed by Mann–Whitney non-parametric test.

Previous studies have shown that exposure to *E. coli*-LF82 reduces epithelial barrier function of T84 cell monolayers.^15^ The decrease in T84 cell monolayer TER that occurred 5 h after infection with *E. coli*-LF82 was partially prevented by IL-10 pre/cotreatment (Figure 4A). Pre/cotreatment with IL-10 significantly prevented apical-to-basolateral passage of *E. coli*-LF82 across T84 epithelial monolayers (Figure 4B and C) and organoids (Figure 4D and E) at 5 h and 11 h postinfection, respectively. ZO-1 immunofluorescence staining revealed that *E. coli*-LF82 infection caused focal disruption of ZO-1 continuity at cell‒cell junctions. IL-10 partially restored the ZO-1 junction organization, whereas IL-10 alone did not significantly alter ZO-1 localization compared to control conditions (Suppl. Figure 3). These findings are consistent with the TER measurements and support a link between mitochondrial preservation and improved epithelial barrier integrity.

### Pre-treatment with IL-10 does not affect IL-8 production evoked by *E. coli*-LF82

Assessing an immune parameter of the epithelial response to *E. coli*-LF82, the increased production of IL-8 observed in infected T84 cells was not affected by IL-10 pre/cotreatment (Suppl. Figure 4). *E. coli*-LF82, which only infected T84 cells, did not produce detectable levels of IL-10, precluding the possibility of a positive feedback loop onto the epithelium. These data indicate that IL-10 preserves epithelial bioenergetics and barrier integrity without impairing IL-8-mediated signaling pathways.

### IL-10 increases phosphorylation of STAT3 at serine-727

**Figure 5.**
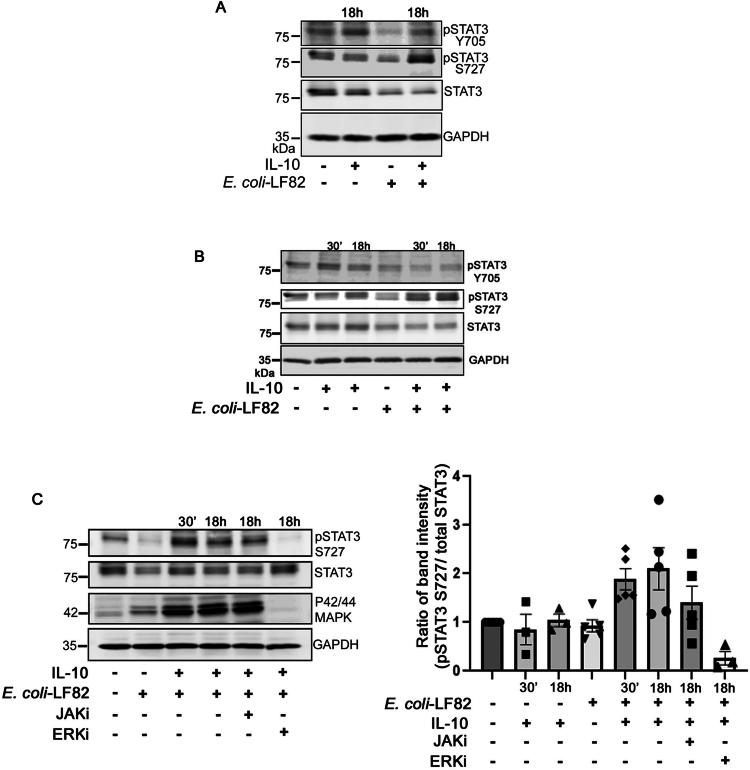
Phospho-STAT3^S727^ is increased in IL-10 primed and *E. coli*-LF82-treated epithelia. Immunoblot analysis of STAT3 phosphorylation in human colonic organoids (A) and T84 cells (B, C) treated for 30 min with IL-10 (10 ng/mL) as a positive control or IL-10 per/cotreated (18 h)+*E. coli*-LF82 (10^8^ CFU/mL, 4 h: MOI = 100). C. Immunoblot analysis of STAT3^S727^ level in T84 cells with either the JAK inhibitor, tofacitinib (10 μM; JAKi) or the ERK p42/44 inhibitor PD-98058 (10 μM; ERKi) for 1 h before IL-10 pre/cotreatment with *E. coli*-LF82. Whole cell lysates (100  µg) were probed with phospho-specific STAT3^S727^, STAT3^Y705^, and p42/44 antibodies and total STAT-3 antibody and GAPDH as a loading control. The blot shown represents one of three blots with similar results. Densitometry data show that pSTAT3^S727^ level is up-regulated in IL-10+*E. coli-*LF82-infected T84 epithelial cells and that this is completely blocked by pretreatment with the ERKi (data are mean ± SEM).

STAT3, as the canonical signaling molecule activated by the IL-10 receptor, was hypothesized to be critical in IL-10 protection against *E. coli-*LF82-induced mitochondrial disruption in epithelia. Epithelia—T84 cells and organoids—exposed to *E. coli*-LF82 for 4 h showed slightly reduced total and *p*-STAT3^Y705^ compared to constitutive levels in non-treated epithelia (Figure 5A and B). Treatment of T84 cells (30 min or 18 h) and colonic organoids (18  h) with IL-10 only resulted in increased *p*-STAT3^Y705^, confirming the expression of functional IL-10 receptors in both preparations (Figure 5A and B). T84 cells and organoids displayed a slightly reduced *p*-STAT3^Y705^ signal with IL-10 + *E. coli*-LF82 treatment compared to IL-10 only (Figure 5A and B). Remarkably, IL-10 + *E. coli*-LF82-exposed cells, but not IL-10-only-treated cells, displayed a marked increase in *p*-STAT3^S727^, a posttranslational modification that promotes STAT3 function in mitochondria.^44-46^ The JAK inhibitor, tofacitinib, completely prevented the IL-10-induced phosphorylation of STAT3^Y705^ (Suppl. Figure 5), but did not reduce the level of phospho-STAT3^S727^ in IL-10+*E. coli*-LF82-treated T84 epithelial cells (Figure 5C). In contrast, pretreatment with the ERK inhibitor, PD-98058 (ERKi), completely blocked activation (*i.e*., phosphorylation) of p42/44 MAPK (also known as ERK1 and ERK2) and the phosphorylation of STAT3^S727^ evoked by IL-10+*E. coli*-LF82 (Figure 5C), suggesting that STAT3^S727^ activation in response to IL-10 treatment in *E. coli*-LF82-infected T84 cells is ERK-dependent (PD-98058 did not affect IL-10-induced phospho-STAT3^Y705^ in T84 epithelia (Suppl. Figure 5)).

Suppressor of cytokine signaling 3 (SOCS3), a well-characterized negative regulator of the STAT3 pathway, can be upregulated in the intestinal epithelium of individuals with Crohn's disease.^47^ Here, *E. coli*-LF82-infected T84 cells showed a significant increase in SOCS3 mRNA levels (Suppl. Figure 6), correlating with the reduced total STAT3 expression (Figure 5B). IL-10 pre/cotreated+ *E. coli-*LF82 infected T84 cells had lower expression of SOCS3 mRNA (Suppl. Figure 6) that could support, at least partially, the observed increase in *p*-STAT3 (Figure 5B and C), suggesting that *E. coli*-LF82-triggered SOCS3 may contribute to dampening of the STAT3 pathway.

**Figure 6.**
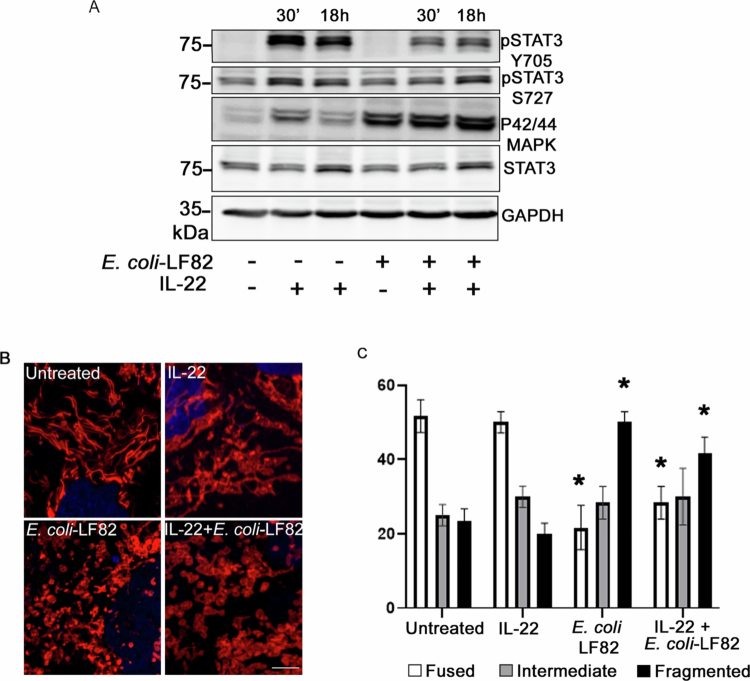
IL-22 does not prevent *E. coli-*LF82-induced mitochondrial fragmentation and activates STAT3 differentially. (A) Immunoblot analysis of whole cell lysates from T84 cells treated with IL-22 (10 ng/mL) for 30  min or 18 h ± *E. coli*-LF82 (10^8^ CFU/mL, 4 h, MOI = 100), reveals increased phospho-STAT3^Y705^ and phospho-ERK1/2 (p42/44) but limited induction of phospho-STAT3^S727^ (see Suppl. 1 A for treatment paradigm). Assessment of mitochondrial morphology by confocal microscopy (B, representative images; scale bar = 1 μm) and semiquantitative analysis of the mitochondrial network (C) in T84 cells pre/cotreated with IL-22 and infected with *E. coli*-LF82 revealed no protective effect of the cytokine (data are mean ± SEM; *n* = 6 epithelial monolayers from three independent experiments. *, *p* < 0.05, compared to untreated cells analyzed by two-way analysis of variance (ANOVA) followed by the Tukey multiple comparison test.

While another member of the IL-10 family, IL-22, caused robust phosphorylation of STAT3^Y705^ in T84 cells using the same treatment regime as IL-10 (Figure 6A), there was, at best, only a subtle increase in phospho-STAT3^S727^ (Figure 6A). Moreover, the mitochondrial fragmentation induced by *E. coli*-LF82 was not rescued by pre/cotreatment with IL-22 (Figure 6B and C), while intriguingly, ERK1/2 phosphorylation was increased in *E. coli*-LF82 ± IL-22-treated T84 cells (Figure 6A).

### Inhibition of p42/44 ERK activity prevents IL-10 protection of mitochondrial function in *E. coli*-LF82-infected T84 cells

**Figure 7.**
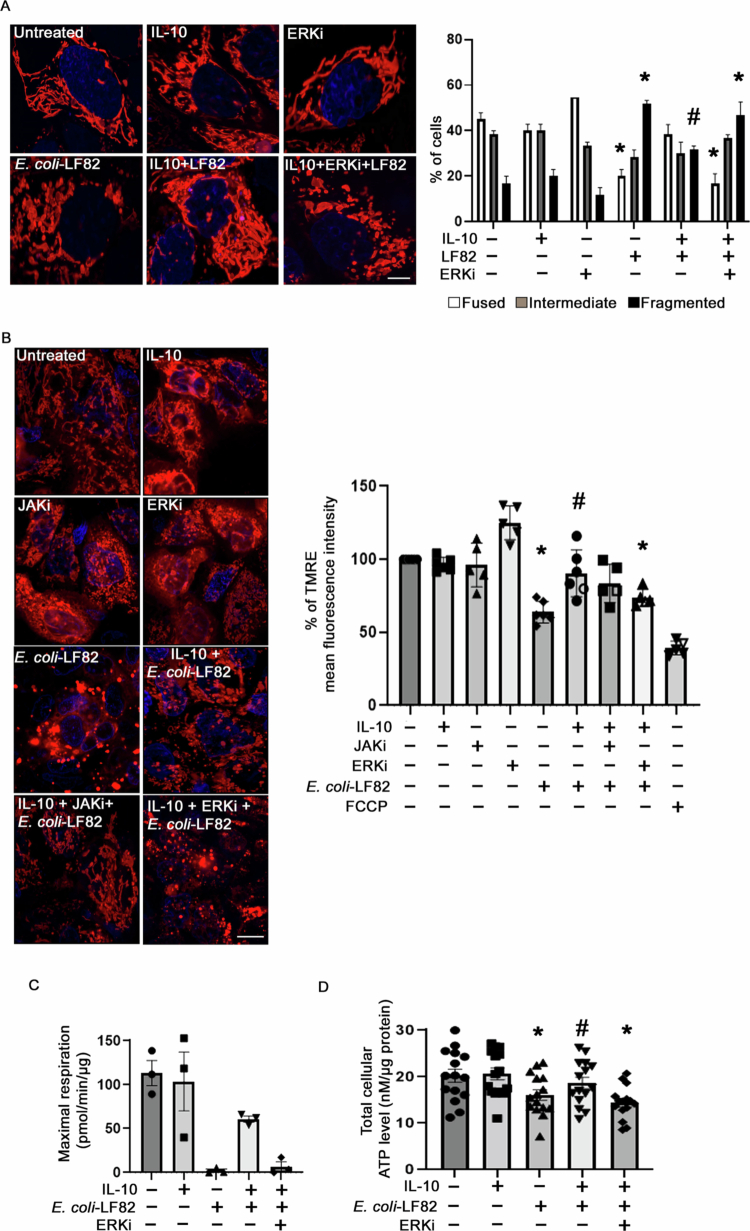
IL-10 preservation of mitochondrial structure is dependent on ERK activity. T84 epithelial cells were treated with the ERK p42/44 inhibitor, PD-98058 (10 μM; ERKi) (or the JAK inhibitor, tofacitinib (10 μM)) for 1 h prior to exposure to pre/cotreatment IL-10 (10 ng/mL, 18 h) followed by *E. coli*-LF82 infection (10^8^ CFU/mL, 4 h, MOI = 100) and then mitochondria were assessed. (A) Representative confocal images of MitoTracker-stained cells and semiquantitative analysis of fragmentation. (B) Representative confocal images of TMRE (400 nM) staining and quantification of mean fluorescence intensity using flow cytometry (*n* = 6 epithelial monolayers from 6 independent experiments. Scale bar = 5 µm). (C) Mitochondrial respiration was assessed in the Seahorse XFe24 analyzer and the quantification of maximal respiration was calculated (*n* = 3 epithelial preparations from independent experiments). (D) Measurement of total cellular ATP levels in T84 cells (*n* = 15 epithelial monolayers from five independent experiments) (data are mean ± SEM; * and #, *p* < 0.05 compared to control untreated cells and *E. coli*-LF82-infected cells, respectively, by one- or two-way ANOVA followed by Tukey's multiple comparison or the Kruskal‒Wallis test followed by Dunn's multiple comparison).

Treatment of T84 epithelia with the ERK inhibitor, but not the JAK inhibitor, counteracted the effect of IL-10 in preserving mitochondrial network structure (Figure 7A), mitochondrial membrane potential (Figure 7B), oxidative phosphorylation (Figure 7C), and ATP levels (Figure 7D) in *E. coli*-LF82 cotreated cells. The JAK or ERK inhibitors alone evoked no changes in mitochondrial form or function (Figure 7A and B).

### IL-10 promotes mitochondrial STAT3^S727^ in AIEC-infected epithelial cells

**Figure 8.**
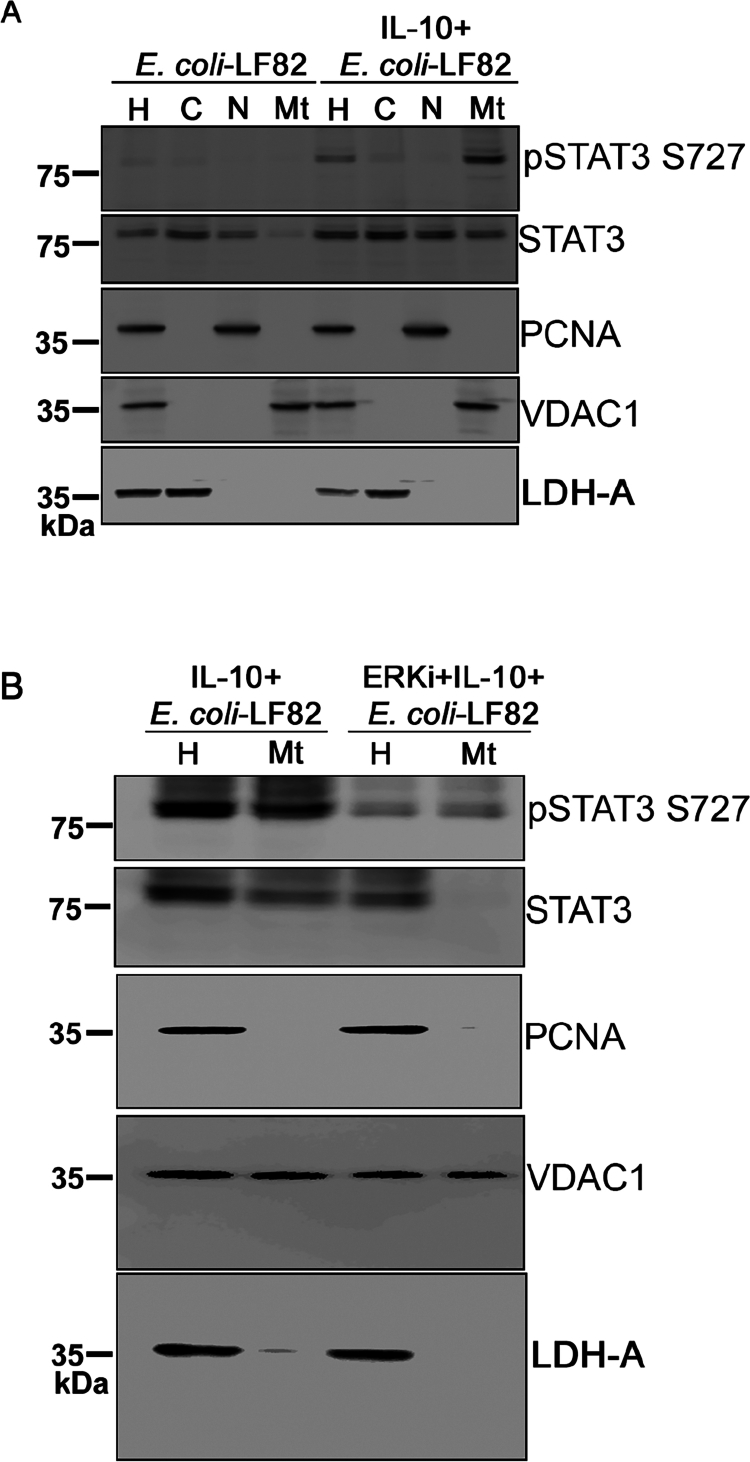
Mitochondrial enrichment of phospho-STAT3^S727^ by IL-10 in AIEC-infected epithelial cells. Subcellular fractionation and immunoblot analysis were performed on *E. coli* LF82-infected (10^8^ CFU, 4 h) T84 cells ± a pre/co-treatment with IL-10 (10 ng/mL,18 h) (A) and with the ERK inhibitor (i), PD-98058 (10 μM; 1 h before the IL-10 treatment). (B). Immunoblot shows phosphorylated STAT3^S727^ in the mitochondrial fraction of IL-10-treated cells, but not in cells infected with *E. coli*-LF82 alone. Fraction purity was validated using PCNA (nuclear marker), VDAC1 (mitochondrial marker), and LDH-A (cytosolic marker). Blot shown represents one of three blots with similar results (H, whole cell extract; C, cytosolic fraction; Mt, mitochondrial fraction; N, nuclear fractions).

STAT3 can localize to mitochondria to regulate electron transport chain activity and cellular metabolism.^25^ To investigate the mitochondria pool of STAT3 following IL-10 treatment, subcellular fractionation of *E. coli*-LF82-infected T84 cells was performed. Fraction purity was confirmed by immunoblotting for PCNA (nuclear marker), VDAC1 (mitochondrial marker), and LDH-A (cytosolic marker). Immunoblot analysis demonstrated an enrichment of *p*-STAT3^S727^ in the mitochondrial fraction of IL-10-treated+*E. coli*-LF82-infected cells that was not observed with *E. coli*-LF82 alone (Figure 8A (lane 4)). Treatment with the ERKi substantially reduced the mitochondrial pool of *p*-STAT3^S727^ in IL-10-treated+*E. coli*-LF82-infected cells (Figure 8B). IL-10 alone induces a modest but detectable level of STAT3 mitochondrial localization (Suppl. Figure 7), indicating that it can prime mitochondrial STAT3 signaling even in the absence of infection. However, the extent of mitochondrial STAT3 accumulation is significantly enhanced during *E. coli*-LF82 infection, suggesting that infection-derived stress signals further promote STAT3 recruitment to mitochondria.

### STAT3^S727^ phosphorylation is required for IL-10-mediated mitochondrial protection in AIEC-infected epithelial cells

**Figure 9.**
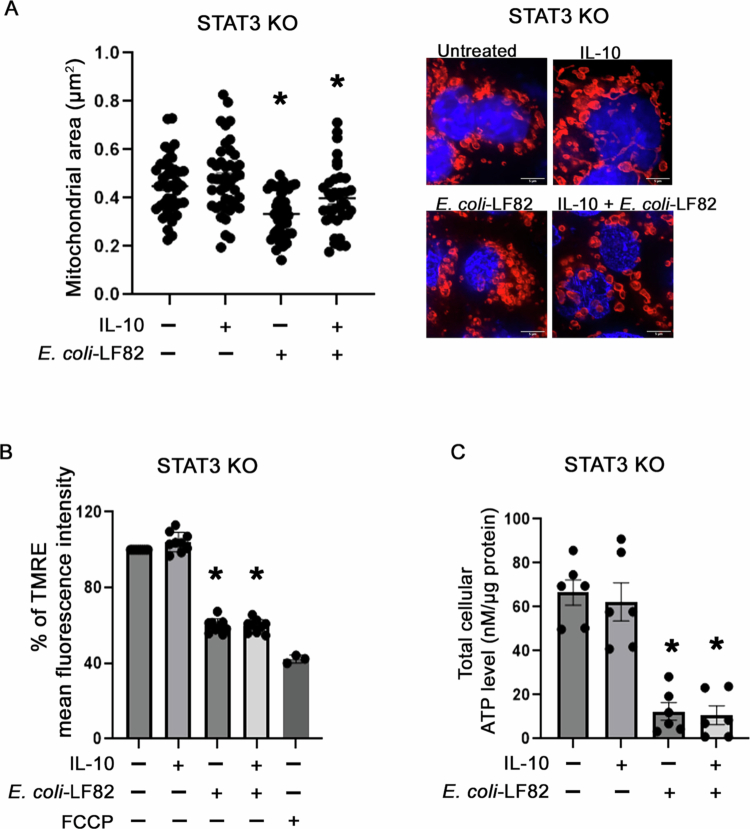
Loss of STAT3 impairs IL-10's ability to preserve mitochondrial function during infection with AIEC. (A) Quantification of mitochondrial area in STAT3 knockout (KO) HT-29 cells. The cells were immunostained with TOMM20 and DAPI and analyzed using a ZEISS microscope (right panel). Mitochondrial area is calculated using Image J applied to randomly selected images of the epithelial layer (*n* = 6 epithelial monolayers (20 cells/monolayer) from 3 independent experiments). (B) Quantification of mitochondrial membrane potential in STAT3 KO cells by TMRE mean fluorescence intensity using flow cytometry. Pre/cotreatment with IL-10 (10 ng/mL, 18 h) did not prevent mitochondrial depolarization induced by *E. coli*-LF82 (10^8^ CFU, 4 h) (*n* = 9 epithelial preparations from 3 independent experiments). (C) Total cellular ATP levels in STAT3 KO cells. (*n* = 6 epithelial monolayers from 3 independent experiments) (Data are mean ± SEM. Mitochondrial area and ATP levels were compared with One-way ANOVA followed by Tukey's multiple comparisons test. The Kruskal‒Wallis test followed by Dunn's multiple comparisons test were used to analyze the TMRE data).

**Figure 10.**
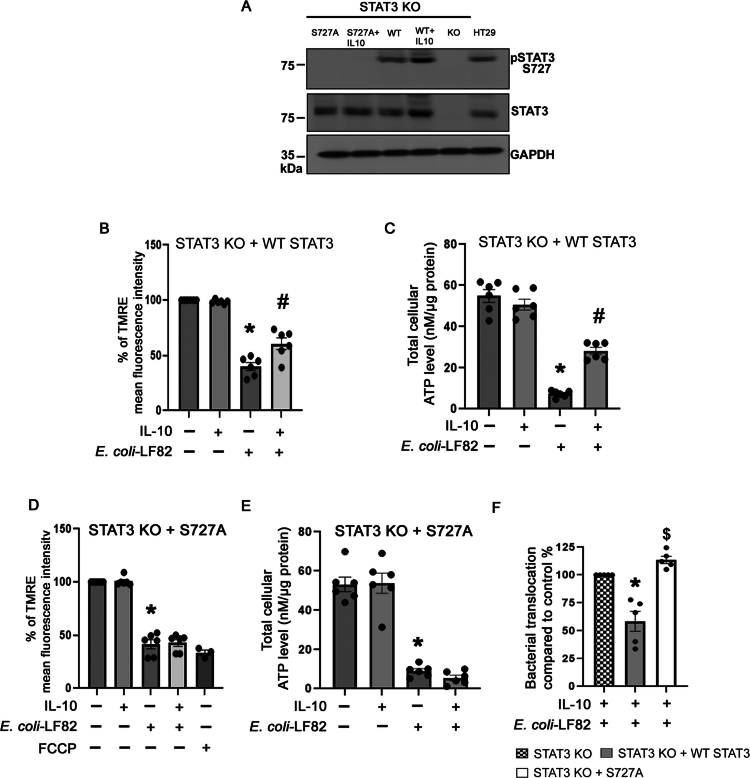
Wild-type STAT3 re-expression rescues IL-10-driven mitochondrial function during AIEC infection, whereas the phospho-mutant STAT3 fails to do so. (A). Immunoblot analysis confirming re-expression of wild-type STAT3 (WT-STAT3) or the phosphorylation-deficient STAT3^S727A^ mutant in STAT3 KO cells via lentiviral transduction. Whole-cell lysates (100  µg) were probed for *p*-STAT3^S727^, total STAT3, and GAPDH (loading control). (B) and (C) are STAT3 KO HT-29 cell reconstituted with WT-STAT3. Cells were pre/co-treated with IL-10 (10 ng/mL) + *E. coli*-LF82 (10^8^ CFU, 4 h) and TMRE fluorescence intensity was measured by flow cytometry to assess mitochondrial membrane depolarization and ATP levels were measured by luminescence assay (*n* = 6 epithelial preparations from 3 independent experiments). (D) and (E) show TMRE analysis and ATP measurement in STAT3 KO HT-29 cells reconstituted with a phosphorylation-deficient STAT3^S727A^ mutant (*n* = 6 epithelial preparations from 3 independent experiments). (F) IL-10 treatment significantly decreased transepithelial translocation of *E. coli-*LF82 across STAT3 KO HT29 cell monolayers reconstituted with WT but not the STAT3^S727A^ mutant (*n* = 5 five independent experiments, 2 monolayers per treatment/experiment) (data are mean ± SEM; *, #, and $, *p* < 0.05 compared to control untreated cells, *E. coli*-LF82-infected cells and WT-STAT3-reconstituted cells, respectively. Statistical analyzes performed were the Kruskal–Wallis test followed by Dunn's multiple comparisons for TMRE data, a one-way ANOVA followed by Tukey's multiple comparisons test for ATP levels and the Mann‒Whitney nonparametric test for the bacterial translocation data.

Postulating that induction of *p*-STAT^S727^ was critical to IL-10's inhibition of *E. coli*-LF82 evoked epithelial mitochondrial disruption, STAT3 knockout (KO) HT-29 epithelial cells were generated using CRISPR/Cas9 technology (Suppl. Figure 8) [the human colon-derived HT-29 cell line, which shows fragmentation of the mitochondrial network after exposure to *E. coli*-LF82,^15^ was used because T84 cells have multiple gene copies of the STAT3 gene that prevented generation of a complete STAT3 knockout T84 cell line]. In HT-29 KO cells, IL-10 did not prevent *E. coli*-LF82–induced mitochondrial dysfunction as measured by mitochondrial area (Figure 9A), depolarization (Figure 9B), or ATP depletion (Figure 9C). Subsequently, the STAT3-KO cells were reconstituted with either wild-type (WT) STAT3 or a STAT3^S727A^ phosphorylation-deficient mutant through retroviral transduction (Figure 10A). Re-expression of WT-STAT3 in the KO cells partially restored the ability of IL-10 to mitigate *E. coli*-LF82-induced mitochondrial depolarization (Figure 10B) and ATP depletion (Figure 10C). In contrast, STAT3-KO cells reconstituted with the STAT3^S727A^ mutant failed to recover mitochondrial function upon IL-10 pre/cotreatment (Figure 10D and E). Similarly, re-expression of WT-STAT3 rescued the IL-10-mediated inhibition of apical-to-basolateral passage of *E. coli*-LF82 across HT-29 epithelial monolayers, which was not observed in IL-10 pre/cotreated STAT3-KO HT-29 cells reconstituted with STAT3^S727A^ (Figure 10F). The partial rescue observed following WT-STAT3 reconstitution likely reflects the heterogeneous nature of the retrovirally transduced population in the absence of clone selection, rather than incomplete functional activity of WT-STAT3. These results indicate that phosphorylated STAT3^S727^ is critical for IL-10-mediated protection of mitochondrial function and epithelial barrier integrity during *E. coli*-LF82 infection.

## Discussion

The intestinal epithelium serves as a selectively permeable barrier, regulating interactions between host immunity and luminal microbes.^48^ We reported that impaired mitochondrial function promoted translocation of commensal bacteria across epithelial cell layers.^7^ In addition, the adherent-invasive *E. coli*-LF82, but not commensal *E. coli*, induced fragmentation of the mitochondrial network in human colonic organoids and epithelial cell lines.^15^ These data, and others,^3^^,^^49^^,^^50^ including demonstration of epithelial mitochondrial fragmentation in inflamed colonic biopsies from individuals with ulcerative colitis,^10^ implicate mitochondrial dysfunction as a participant in enteric inflammation.^51-53^ The colitis observed in *il-10*^−/−^ mice^21^ and the devasting very early-onset IBD in children with dysfunctional IL-10 receptors highlight IL-10's pivotal roles in limiting inflammation.^22^^,^^54^^,^^55^ Assessment of murine macrophages revealed that IL-10 influences oxidative phosphorylation, mitophagy, and mitochondrial dynamics.^30^^,^^33^^,^^35^ Therefore, given its ability to influence bioenergetics and importance in immunoregulation, we hypothesized that IL-10 might protect against *E. coli*-LF82 evoked epithelial mitochondrial dysfunction, the concomitant reduction in barrier integrity and increased chemokine production.

The mitochondrial fragmentation observed in *E. coli*-LF82-infected human organoids and T84 epithelial cells were almost completely prevented by exposure to IL-10 18 h prior to and along with infection, to mimic IL-10 availability in a healthy gut. The reduced mitochondrial membrane potential (ΔΨ_M_) and opening of the mitochondrial permeability transition pore (mPTP) in *E. coli*-LF82-infected cells were reduced by IL-10, suggesting that mPTP opening and mitochondrial fragmentation are likely interconnected events.^56^ Similarly, IL-10 promoted mitochondrial “health” (i.e., increased ΔΨ_M_) in Caco2 epithelia^35^ and murine macrophages^57^ exposed to LPS: neither study assessed mitochondrial dynamics.

Functional studies revealed shutdown of oxidative phosphorylation (OxPhos) as gauged by a reduced OCR, and a concomitant, although milder reduction in ATP levels in *E. coli*-LF82-infected T84 cells. Although *E. coli*-LF82 infection markedly impairs maximal mitochondrial respiration, total cellular ATP levels are only partially reduced, likely reflecting compensatory metabolic adaptation through increased reliance on glycolysis. These bioenergetic deficits were accompanied by reduced epithelial barrier function and increased translocation of the *E. coli* across the epithelial layer. Increased epithelial permeability is a common feature of infection with microbial pathogens.^58-60^ Suppression of OxPhos and reduced ATP levels, and/or a shift to glycolysis (i.e., Warburg effect) have been observed with many bacterial pathogens and effector molecules,^61^ such as in *Legionella pneumophila* or *Mycobacterium tuberculosis* infected macrophages.^62^^,^^63^ While it is unclear if the reduced OxPhos and ATP levels are a host antimicrobial response or are of benefit to the survival and transmission of the pathogens, other bacteria, such as *Chlamydia trachomatis* and *Lactobacillus gasseri,* can stimulate increased ATP production and OxPhos,^64-66^ underscoring the specificity of the host cell type-bacterial species interaction.^18^ The ability of IL-10 to partially normalize the *E. coli*-LF82-induced bioenergetic and barrier defects highlights the importance of IL-10 in the regulation of enteric epithelial‒bacterial interactions and are consistent with the increased susceptibility of *il-10*^−/−^ mice to AIEC.^57^ While not the focus here, it is noteworthy that the *E. coli*-LF82 evoked epithelial IL-8 production was not affected by IL-10, revealing refinement and selectivity in IL-10's amelioration of the impact of AIEC on T84 cells. Indeed, the sustained production of IL-8 *in vivo* to recruit neutrophils would be an important component of the host's response to the pathobiont.

An indirect explanation for the effect of IL-10 would be inhibition of bacterial growth or increased epithelial killing of the *E. coli*. However, IL-10 was neither directly bacteriostatic nor bactericidal, and there was no evidence that IL-10 up-regulated a T84 cell anti-bacterial response against *E. coli*-LF82.

IL-10-induced phosphorylation of STAT3^Y705^ causes dimerization and translocation to the nucleus to affect gene transcription, while phosphorylation at STAT3^S727^ has been associated with mitochondrial function.^23-25^^,^^44^ Accordingly, we observed increased *p*-STAT3^Y705^ and *p*-STAT3^S727^ in IL-10-treated T84 cells. There was enhancement of *p*-STAT3^S727^, but not *p*-STAT3^Y705^, after 18 h of IL-10 exposure and *E. coli*-LF82 infection, compared to IL-10 alone (*E. coli*-LF82 only did not evoke a *p*-STAT3^S727^ signal). ERK1/2, among other kinases, can phosphorylate STAT3 at S727, linking MAPK activation to non-canonical STAT3 signaling.^67^ Enteropathogenic *E. coli* (EPEC) can activate ERK signaling,^37^ and we observed increased *p*-ERK1/2 levels following infection with *E. coli*-LF82 that was more marked in IL-10 co-treated T84 cells and completely ablated by the ERK-inhibitor, PD-98058, but not inhibition of JAK activity (which did reduce *p*-STAT3^Y705^). Pharmacological inhibition of ERK1/2 activation blocked the IL-10+*E. coli*-LF82 induced *p*-STAT3^S727^ and negated the ability of IL-10 to preserve mitochondrial network integrity and ΔΨ_M_ in the face of challenge with *E. coli*-LF82. ERK- and JAK-dependent signaling differentially influence mitochondrial structure and membrane potential, with ΔΨ_M_ reflecting not only network integrity but also broader metabolic and signaling inputs, likely accounting for the apparent divergence between morphological and functional readouts.

While others have shown that interaction between STAT3 and mTOR mediates some effects of IL-10,^30^ the data herein indicate that ERK1/2 activity is critical for IL-10-evoked *p*-STAT3^S727^ and maintenance of mitochondrial form and function. This finding is in accordance with a hepatocyte growth factor-induced ERK-*p*-STAT3^S727^-mitochondrial pathway that promoted neurite outgrowth.^68^ The synergistic interaction of IL-10+*E. coli*-LF82 in the heightened expression of epithelial *p*-STAT3^S727^ is intriguing, but as yet unexplained. Perhaps IL-10 plays a priming role by initiating STAT3 activation, inducing a conformational change that renders the protein a more suitable substrate for ERK-driven phosphorylation of S727. *E. coli*-LF82-induced ERK signaling could facilitate the spatial and temporal coordination required for efficient *p*-STAT3^S727^. Alternatively, disruption of endogenous phosphatases and regulators of STAT activity (e.g., SOCS3 and PIAS1) could sustain the *p*-STAT3^S727^ signal. Our findings demonstrate that *E. coli*-LF82 infection is associated with reduced STAT3 protein abundance and increased SOCS3 expression in intestinal epithelial cells. While the precise mechanism of the IL-10-ERK-*p*-STAT3^S727^ signaling axis requires elucidation, the data nevertheless highlight the importance of integrated signaling between cytokine and microbial cues in regulating epithelial STAT3 activity beyond its nuclear transcriptional role to maintain mitochondrial homeostasis.

Many chemokines, such as IL-6 family members, IL-23, and notably, IL-22, which can regulate epithelial barrier function, utilize STAT3 for signal transduction.^69^^,^^70^ There is abundant evidence that IL-22-mediated STAT3 activation predominantly drives nuclear gene transcription.^71^^,^^72^ Testing the concept of STAT3 maintenance of the mitochondrial network, IL-22 caused increased *p*-STAT3^Y705^ and a subtle increase in *p*-STAT3^S727^ and both signals were reduced when *E. coli*-LF82 was present. IL-22 treatment did not prevent *E. coli*-LF82-evoked mitochondrial fragmentation, correlating with an inadequacy of the *p*-STAT3^S727^ despite a clear increase in *p*-ERK1/2. These findings suggest that ERK activation alone is insufficient for mitochondrial rescue, and that IL-10 likely provides additional signaling inputs such as PI3K,^73^ sustained STAT3 activation, and engagement of metabolic regulatory pathways that enable effective STAT3 S727 phosphorylation and mitochondrial protection, which are not recapitulated by IL-22.

Phosphorylation of STAT3 at S727 has been implicated in translocation to mitochondria, a process facilitated by interaction with GRIM-19, a component of mitochondrial Complex I.^44^ Cell fractionation studies identified enrichment of *p*-STAT3^S727^ in the mitochondrial fraction of IL-10+*E. coli*-LF82-treated T84 epithelia. While IL-10 is sufficient to initiate mitochondrial translocation of STAT3, this effect is markedly amplified under infectious conditions, suggesting that IL-10 primes mitochondrial STAT3 signaling under basal conditions, whereas infection provides additional stress-dependent cues that enhance its mitochondrial recruitment. Adopting a knockout and replacement strategy, genetic ablation of STAT3 in HT-29 epithelial cells abolished the ability of IL-10 to protect the cells from *E. coli*-LF82 disruption of mitochondrial function. Functional rescue experiments confirmed that re-expression of wild-type STAT3, but not the phosphorylation-deficient S727A mutant, restored IL-10's ability to support mitochondrial function in *E. coli*-LF82-infected HT-29 cells. Collectively, the findings reveal a new immunoregulatory role for IL-10 in the gut, whereby an ERK-*p*-STAT3^S727^-mitochondrial interaction preserves epithelial mitochondrial function upon exposure to the bacterial pathobiont, AIEC (strain LF82). We speculate that this protection could extend to other enteric intracellular pathogens and be relevant to other cell types, such as macrophages.

At the boundary between the external and internal environments, the enteric epithelium plays a critical role in the balance of health and disease and the mitochondrial contribution to this is increasingly apparent. Having shown previously that microbe-derived factors (e.g., butyrate) help preserve epithelial mitochondrial form and function in the context of bacterial pathobiont infection,^16^ the current study demonstrates that IL-10 helps maintain epithelial barrier integrity in a *p*-STAT3^S727^-dependent fashion by limiting AIEC-triggered loss of mitochondrial network structure and function likely due to opening of the mPTP and loss of mitochondrial membrane potential.

## Supplementary Material

Supplementary Material Supplementary_Figure_and_Legends.docx

## Data Availability

The authors confirm that the data supporting the findings of this study are available within the article and its supplementary materials.

## Abbreviations

AIEC: adherent invasive *Escherichia coli*
ATP: adenosine triphosphate
OCR: oxygen consumption rate
TOMM20: translocase of outer mitochondrial membrane 20
ZO-1: zonula occludens-1
FCCP: carbonyl cyanide 4-(trifluoromethoxy) phenylhydrazone
TMRE: tetramethyl rhodamine ethyl ester
CFU: colony-forming unit
IBD: inflammatory bowel disease
IL: interleukin
TER: transepithelial resistance
ERK: extracellular signal-regulated kinase
STAT3: signal transducer and activator of transcription 3
SOCS: suppressor of cytokine signaling
