# Supplementary material for: IL-10-STAT3 axis preserves epithelial mitochondrial homeostasis during bacterial challenge
Source: Gut Microbes. 2026 Apr 23;18(1):2661411. doi: 10.1080/19490976.2026.2661411 (PMC13108364; doi:10.1080/19490976.2026.2661411)
Supplement: Supplementary Material — Supplementary_Figure_and_Legends.docx [file KGMI_A_2661411_SM4027.docx]

**Supplementary Figure and Legends**

**
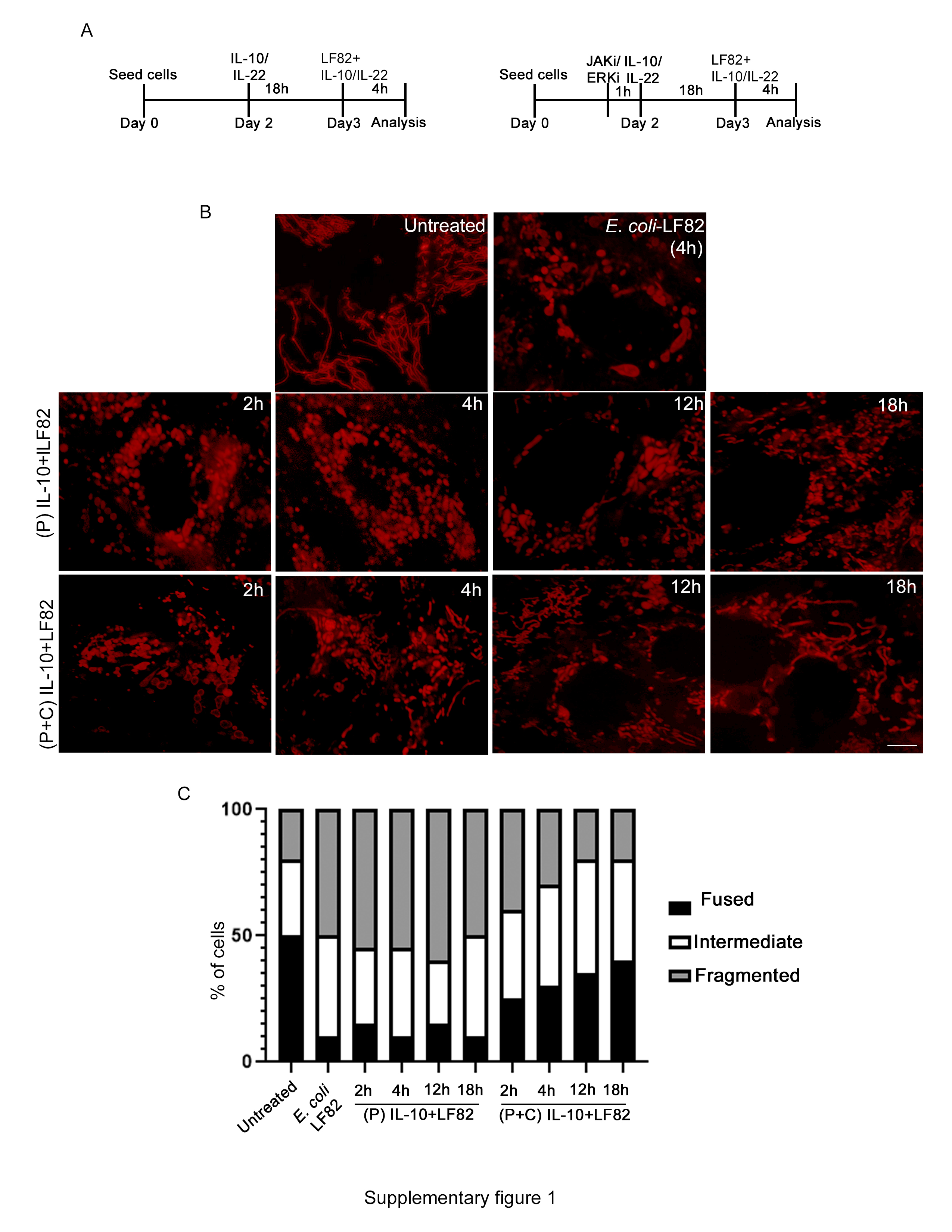
S. Figure 1. (**A) Experimental timeline for treatment of T84 epithelial cells with cytokines (IL-10 or IL-22, each at 10 ng/mL), pharmacological inhibitors (tofacitinib [JAK inhibitor, 10 μM] and PD-98059 [ERK inhibitor, 10 μM]), and *E. coli*-LF82 (multiplicity of infection = 100, 4h). (B) Time course analysis of IL-10 effect on mitochondrial morphology during *E. coli*-LF82 infection. T84 cells were pre-treated with IL-10 for 2, 4, 12 or 18h ± co-treatment with *E. coli*-LF82 and stained with MitoTracker red and Hoest dye. The images were acquired using a ZEISS microscope under 60x oil immersion. Scale bar = 5 µm. (P) IL-10 + LF82 indicates pre-treatment with IL-10 prior to *E. coli*-LF82 exposure, whereas (P+C) IL-10 + LF82 indicates pre-treatment followed by co-treatment with IL-10 during *E. coli*-LF82 exposure. (C) Quantification of fused, intermediate, and fragmented mitochondria in T84 cells revealed that co-treatment of IL-10 is required to prevent *E. coli*-LF82 induced mitochondrial fragmentation (n=3 epithelial preparations/condition from 1 experiment).

**
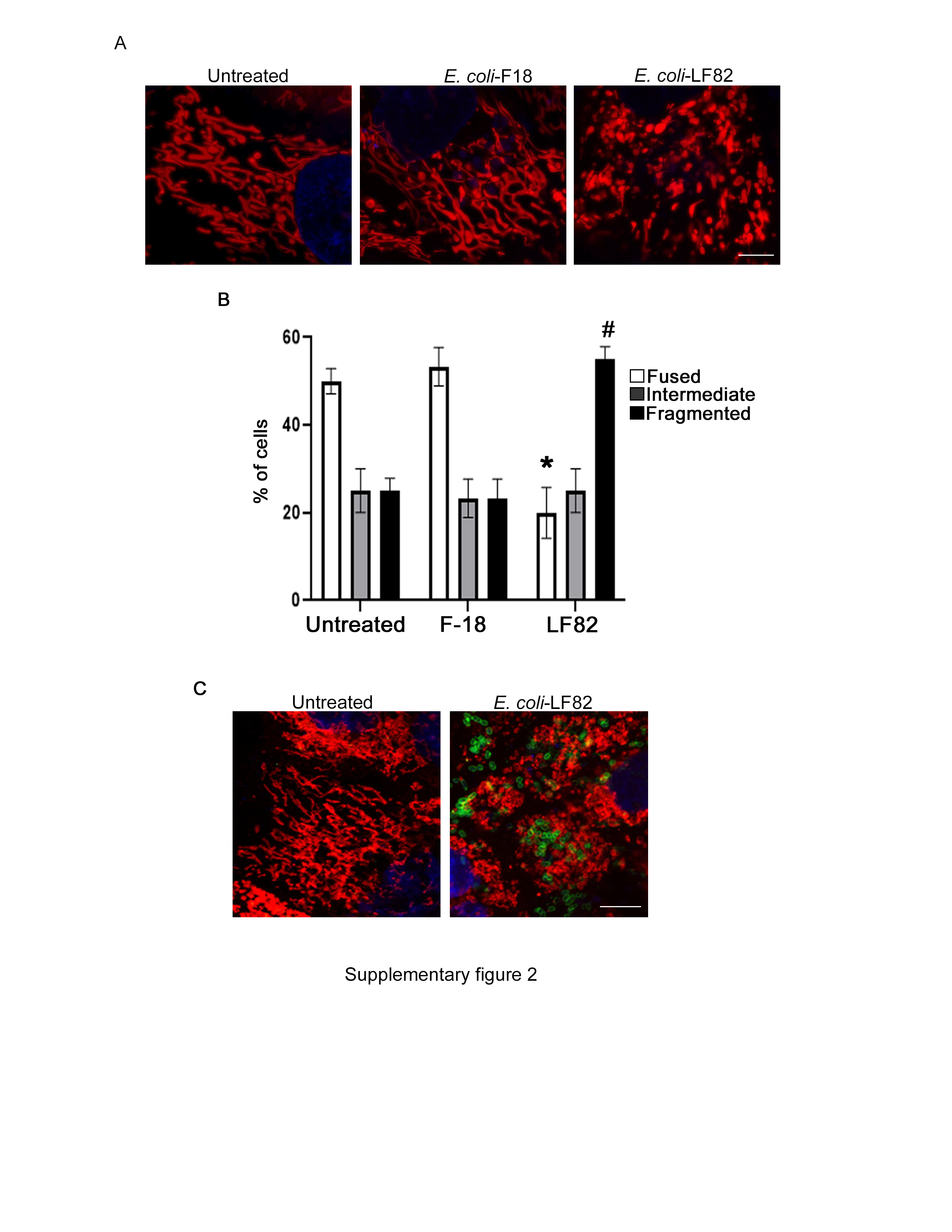
S. Figure 2. The AIEC pathobiont, *E. coli*-LF82, induces mitochondrial fragmentation in epithelial cells. (**A) Analysis of mitochondrial morphology by confocal microscopy and quantification (B) of fused, intermediate and fragmented mitochondria networks show that *E. coli*-LF82 (10^8^ CFU/mL, 4h), but not commensal bacteria *E. coli*-F18 (10^8^ CFU/mL, 4h) induces mitochondrial fragmentation in T84 epithelial cells. Scale bar = 1 μm (data are mean ± SEM; n=6 epithelial monolayers from three independent experiments; * and #, p<0.05 compared to control untreated cells by two-way ANOVA followed by the Tukey multiple comparison test). (C) Immunofluorescence of T84 cells treated with *E. coli*-LF82 show the bacteria (green) in cells with a fragmented mitochondria network (red). Scale bar = 5 μm.

**
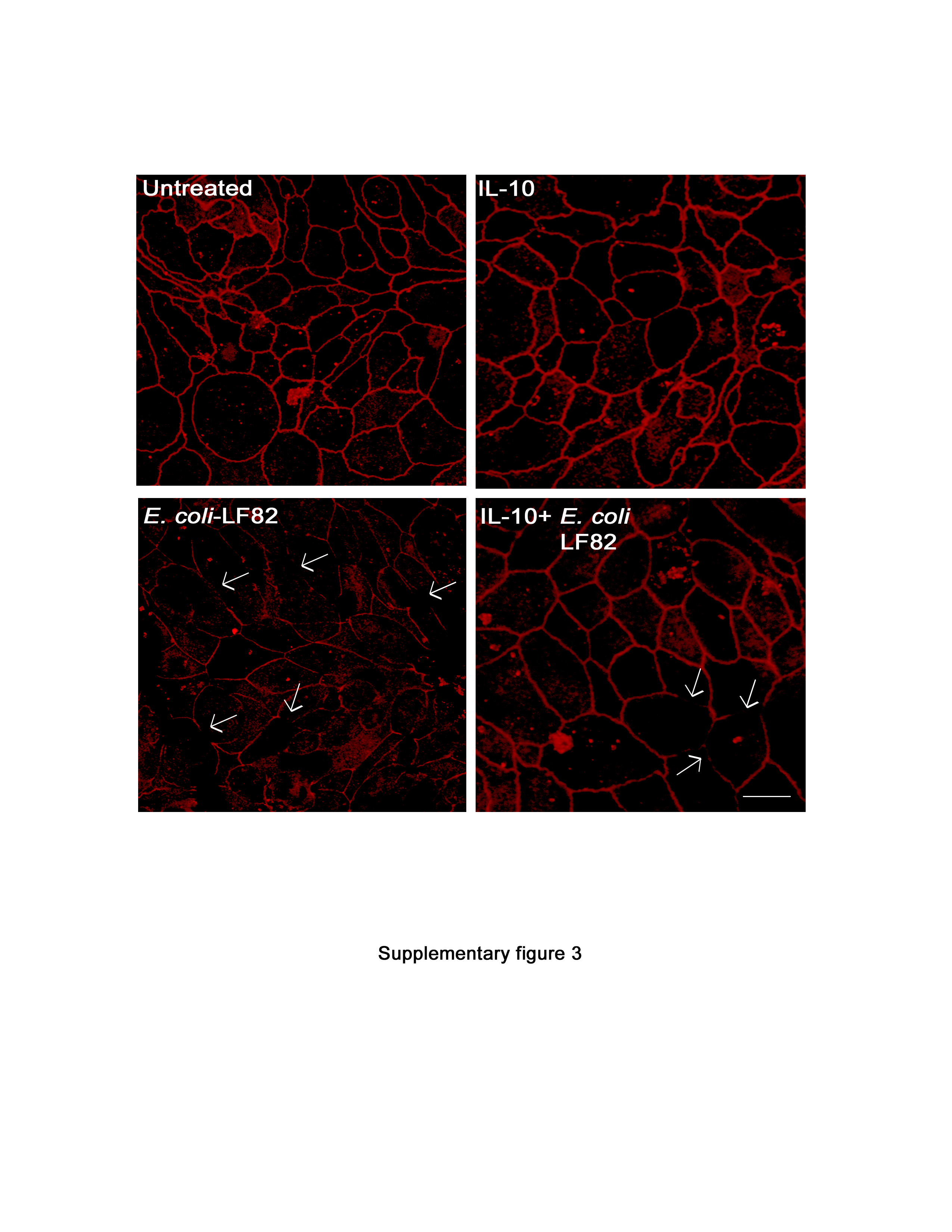
S. Figure 3. IL-10 restores ZO-1 continuity at tight junction. (**A) ZO-1 immunofluorescence analysis of T84 epithelial monolayers infected with *E. coli*-LF82 (MOI=100; 4h) disrupted ZO-1 continuity at tight junctions (arrows), while IL-10 (10 ng/mL) pre/co-treatment partially restores junctional organization. Scale bar = 20μm.

**
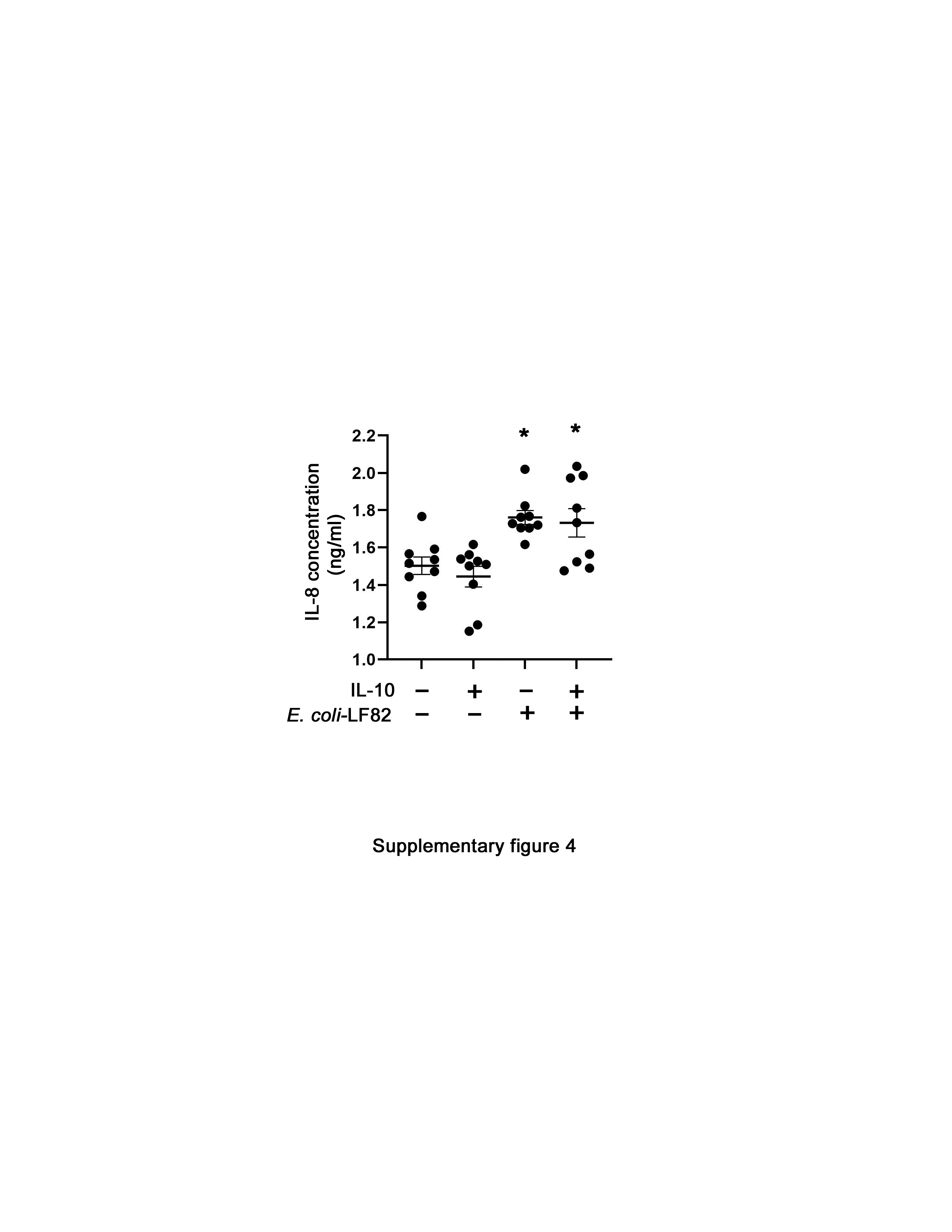
**

**S. Figure 4. IL-10 does not affect IL-8 production by *E. coli*-LF82 infected epithelia.** T84 epithelial cell monolayers exposed to *E. coli*-LF82 (MOI=100; 4h) showed increased IL-8 protein levels, which were not affected by pre/co-treatment with IL-10 treatment (10 ng/mL) (data are mean ± SEM; n=9 epithelial monolayers from 3 independent experiments; *, p<0.05 compared to control untreated cells by One-way ANOVA followed by the Tukey’s multiple comparison test).

**
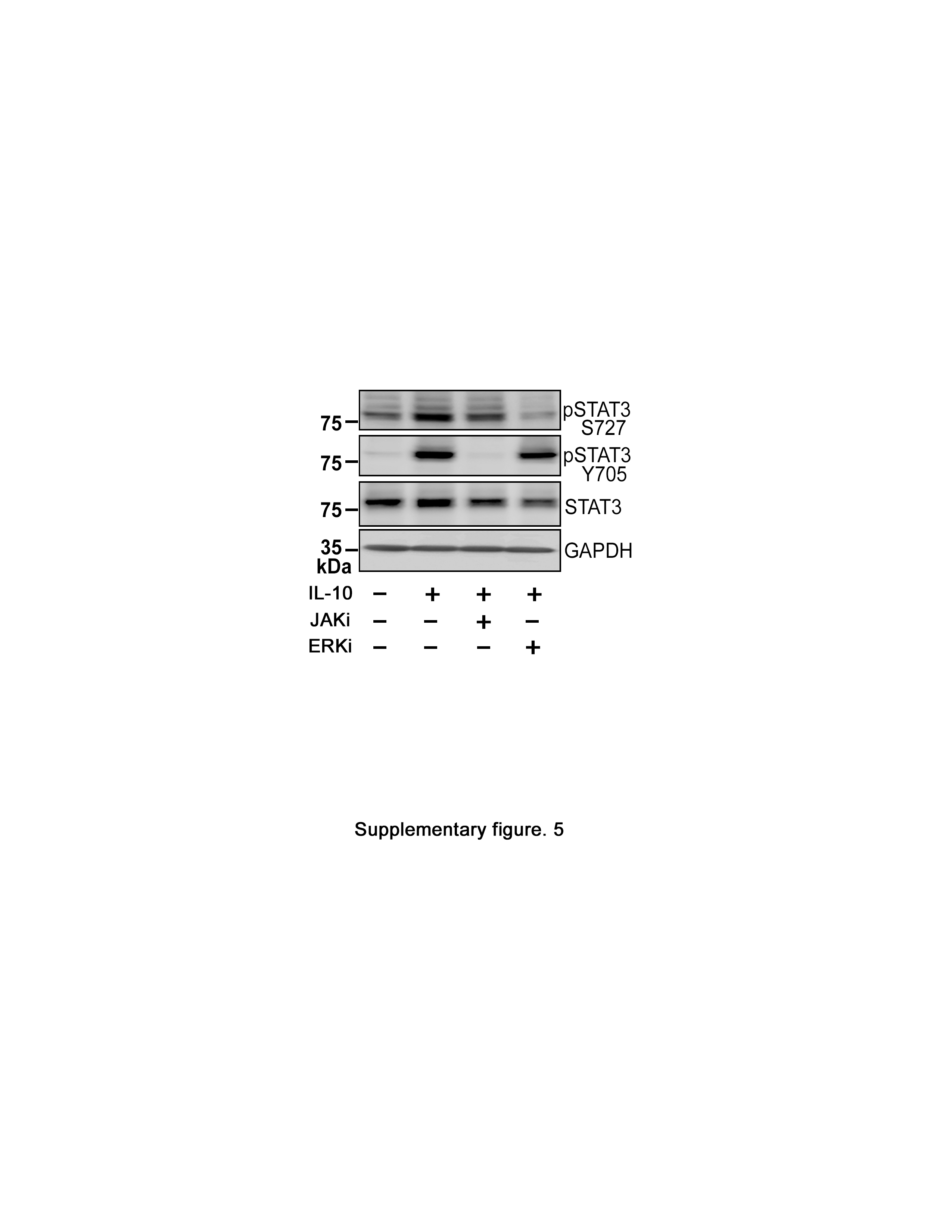
S. Figure 5. Tofacitinib and PD-98058 activity confirmed.** Westen blot analysis of STAT3 phosphorylation in T84 epithelial cells treated with IL-10 (10 ng/mL for 30 min) ± the JAK inhibitor, tofacitinib (10 μM; JAKi) or the ERK inhibitor, PD-98058 (10 μM; ERKi), 1h before addition of IL-10. Protein in whole cell lysates (100 µg) was probed with antibodies selective for phospho-STAT3^S727^, phospho-STAT3^Y705^, total STAT3 and GAPDH as an additional loading control. Activity of the pharmacological inhibitors of JAK and ERK was confirmed by their capacity to reduce IL-10 evoked phospho-STAT^Y705^ and phospho-STAT^S727^, respectively.

**
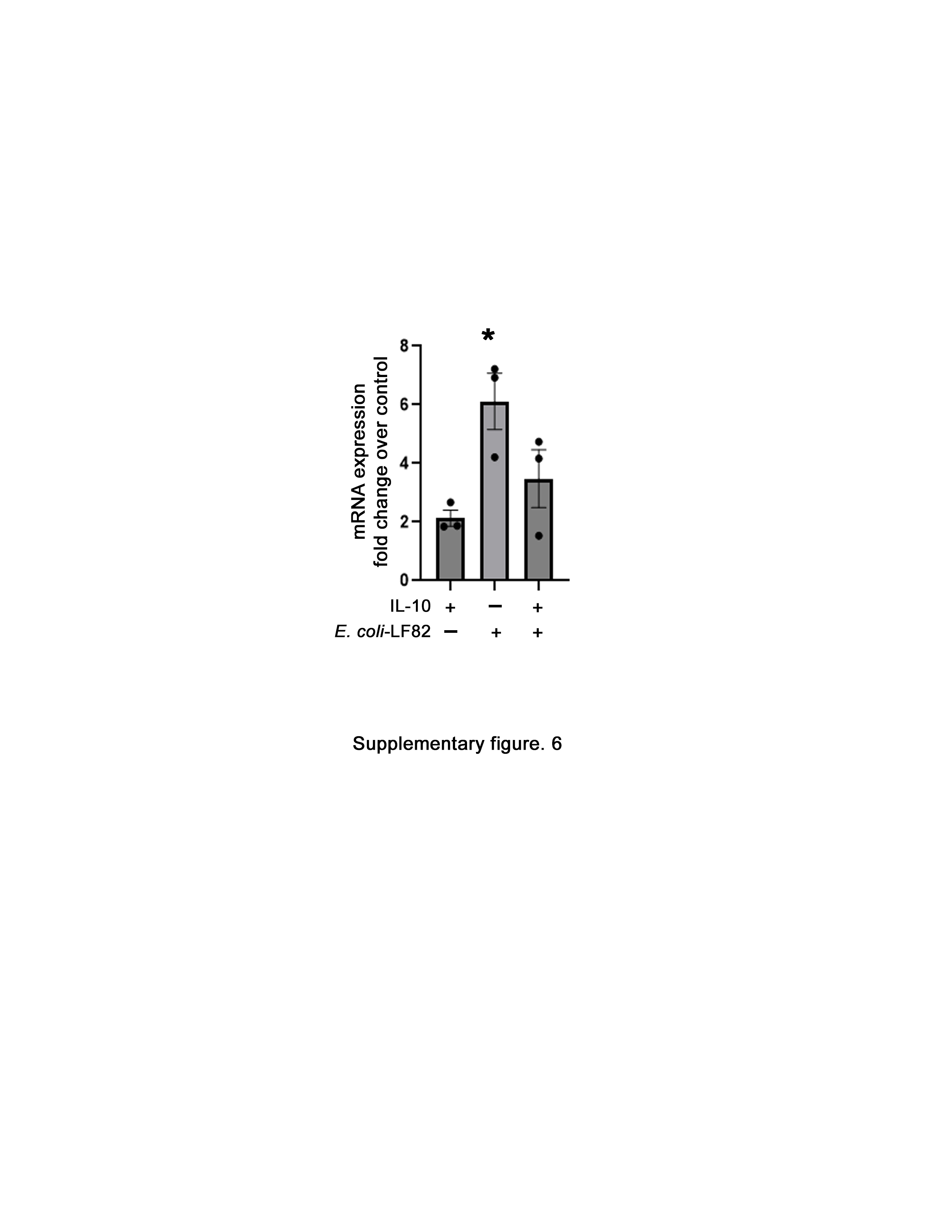
S. Figure 6. IL-10 modulates SOCS3 expression in intestinal epithelial cells during *E. coli*-LF82 infection.** T84 epithelial cells were infected with *E. coli* LF82 (MOI=100; 4h) in the presence or absence of IL-10 (10 ng/mL, 18h) and SOCS3 mRNA levels were measured by qPCR. *E. coli*-LF82 infection significantly upregulated SOCS3 expression, whereas IL-10+ *E. coli*-LF82 reduced SOCS3 levels. Data represent mean ± SEM of three independent experiments. *, p<0.05 compared to control untreated cells by One-way ANOVA followed by the Kruksal-wallis comparison test.

**
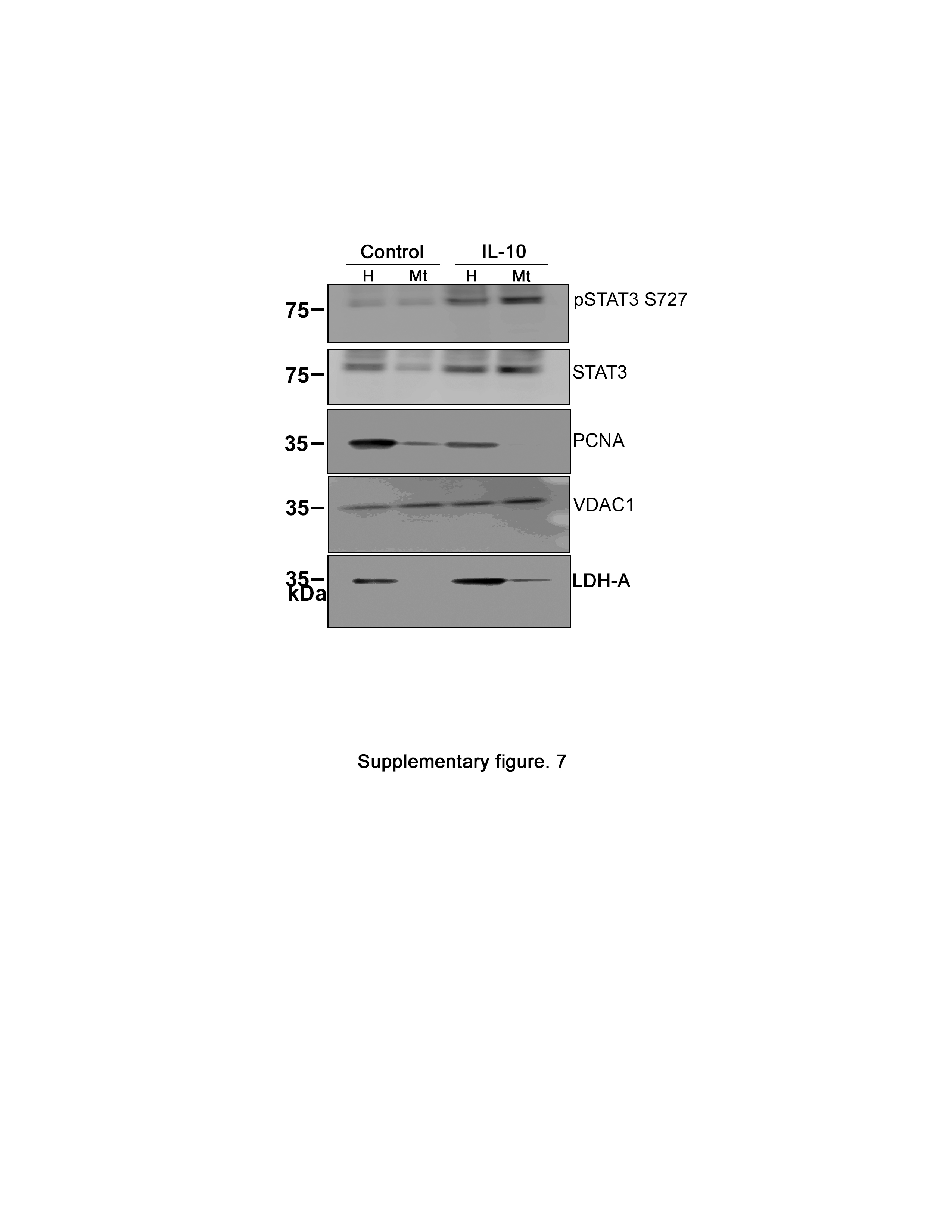
S. Figure 7.** **IL-10 can independently induce mitochondrial STAT3 translocation.** Subcellular fractionation and immunoblot analysis were performed on T84 cells treated with IL-10 (10 ng/mL, 30min). Fraction purity was validated using PCNA (nuclear marker), VDAC1 (mitochondrial marker), and LDH-A (cytosolic marker). (H, whole cell extract; Mt, mitochondrial fraction).

**
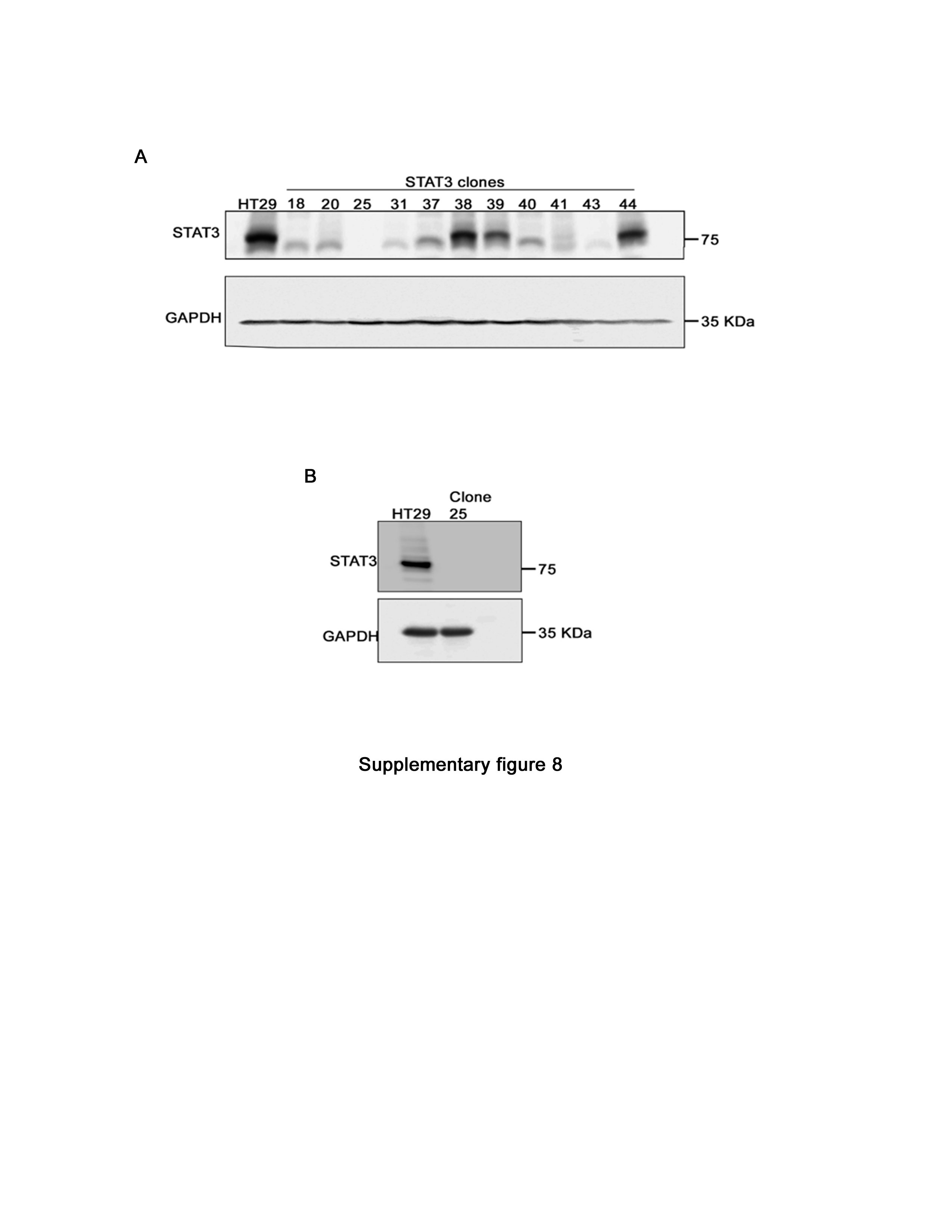
S. Figure 8. STAT3 gene knock out confirmation by Western blot analysis.**

(A). CRISPR-Cas9 gene knockout clones were screened by western blot using anti-STAT3 antibody. The membranes were also probed with GAPDH as a loading control. Western blot analysis results showed that HT-29 cell clone 25 is a complete STAT3 gene knockout, with clones 18, 20, 31, 37, 40, 41 and 43 having substantial or partial knockout/knockdown of the STAT3 gene. (B). Validation of STAT3 KO clone 25 by western blot analysis.
